# From RGB-D to RGB-Only: Reliability and Clinical Relevance of Markerless Skeletal Tracking for Postural Assessment in Parkinson’s Disease

**DOI:** 10.3390/s26041146

**Published:** 2026-02-10

**Authors:** Claudia Ferraris, Gianluca Amprimo, Gabriella Olmo, Marco Ghislieri, Martina Patera, Antonio Suppa, Silvia Gallo, Gabriele Imbalzano, Leonardo Lopiano, Carlo Alberto Artusi

**Affiliations:** 1 Institute of Electronics, Computer and Telecommunication Engineering (IEIIT), Consiglio Nazionale delle Ricerche (CNR), 10129 Turin, Italy; 2 Department of Control and Computer Engineering, Politecnico di Torino, 10129 Turin, Italy; gabriella.olmo@polito.it; 3 Department of Electronics and Telecommunications and Polito^BIO^Med Lab, Politecnico di Torino, 10129 Turin, Italy; marco.ghislieri@polito.it; 4 Department of Human Neurosciences, Sapienza University of Rome, 00185 Rome, Italy; martina.patera@uniroma1.it (M.P.); antonio.suppa@uniroma1.it (A.S.); 5 IRCCS Neuromed, 86077 Pozzilli, Italy; 6 Department of Neurosciences “Rita Levi Montalcini”, University of Turin, 10126 Turin, Italy; sil.gallo@unito.it (S.G.); gabriele.imbalzano@unito.it (G.I.); leonardo.lopiano@unito.it (L.L.); 7 Department of Neuroscience, Biomedicine and Movement Sciences, University of Verona, 37134 Verona, Italy; carloalberto.artusi@univr.it

**Keywords:** markerless human pose estimation, Parkinson’s Disease, axial symptoms, postural assessment, Google MediaPipe, Microsoft Azure Kinect, clinical validation, technical validation, digital biomarkers, angular measurements

## Abstract

Axial postural abnormalities in Parkinson’s Disease (PD) are traditionally assessed using clinical rating scales, although picture-based assessment is considered the gold standard. This study evaluates the reliability and clinical relevance of two markerless body-tracking frameworks, the RGB-D-based Microsoft Azure Kinect (providing the reference KIN_3D model) and the RGB-only Google MediaPipe Pose (MP), using a synchronous dual-camera setup. Forty PD patients performed a 60 s static standing task. We compared KIN_3D with three MP models (at different complexity levels) across horizontal, vertical, sagittal, and 3D joint angles. Results show that lower-complexity MP models achieved high congruence with KIN_3D for trunk and shoulder alignment (ρ > 0.75), while the lateral view significantly improved tracking of sagittal angles (ρ ≥ 0.72). Conversely, the high-complexity model introduced significant skeletal distortions. Clinically, several angular parameters emerged as robust metrics for postural assessment and global motor impairments, while sagittal angles correlated with motor complications. Unexpectedly, a more upright frontal alignment was associated with greater freezing of gait severity, suggesting that static postural metrics may serve as proxies for dynamic gait performance. In addition, both RGB-only and RGB-D frameworks effectively discriminated between postural severity clusters. While the higher-complexity MP model should be avoided due to inaccurate 3D reconstructions, our findings demonstrate that low- and medium-complexity MP models represent a reliable alternative to RGB-D sensors for objective postural assessment in PD, facilitating the widespread application of objective posture measurements in clinical contexts.

## 1. Introduction

Axial Postural Abnormalities (PAs) are common and critical dysfunctions across various pathologies [1,2,3,4]. They frequently lead to balance deficits, instability, and a significantly increased risk of falls, posing a serious threat to patient safety and independence [5]. While early detection of PA onset serves a crucial preventative function, quantifying changes in existing PAs over time is equally essential for optimizing therapeutic interventions and improving patient quality of life [6].

This need for objective quantification is particularly relevant in Parkinson’s Disease (PD), in which axial PAs are highly prevalent, ranging from frequent subtle flexed postures of the trunk and lower limbs to severe deformities such as camptocormia, antecollis, or Pisa syndrome, which affect over 20% of patients [7]. In fact, subtle medio-lateral or antero-posterior flexion is present even in early phases, requiring continuous compensatory actions for postural control [8]. In addition to altered postural alignment, many patients with PD exhibit postural instability, which manifests as increased body sway during quiet standing and reduced stability during voluntary or externally perturbed movements. This instability reflects the progressive involvement of axial motor control mechanisms and may be further influenced by disease-related factors, the presence of dyskinesias, or fluctuations related to dopaminergic therapy [9].

The current assessment of PA severity predominantly relies on subjective clinical rating scales or analog measuring tools (e.g., goniometers), often lacking precision and inter-rater reliability [10,11,12,13]. While marker-based Motion Capture (MoCap) systems represent the gold standard for accurate and objective measurement, their high cost, time-consuming setup, and strict laboratory requirements limit their use to specialized facilities, making them unsuitable for long-term monitoring or large-scale applications [14]. This has driven the demand for non-invasive, cost-effective technological solutions that provide easy-to-use, quantitative, and repeatable measurements even in unconstrained environments such as home settings [15,16].

Early technological efforts favored wearables for motion analysis, particularly for quantifying postural instability [17,18,19,20,21]. However, wearables face several limitations for comprehensive postural assessment, particularly in older patients and in subjects limited by motor impairment: the need for numerous sensors for multi-segmental analysis, complex calibration and synchronization, and inherent patient discomfort. This has led to the emergence of non-invasive video analysis using body-tracking algorithms. The field evolved from marker-assisted RGB systems, based on pure computer vision techniques, to more sophisticated RGB-Depth (RGB-D) cameras (e.g., Microsoft Kinect), which provide 3D skeletal tracking via depth mapping [22,23,24,25,26,27]. Unfortunately, these specialized RGB-D sensors are plagued by short product lifecycles and planned obsolescence, hindering the long-term viability and large-scale adoption of the applications they enable. This emphasizes the need to transition away from specialized depth-sensing hardware toward more readily available, inexpensive, and stable RGB-only devices (e.g., standard cameras). Moving away from specific, short-lived depth sensors toward simpler, less expensive, and readily available RGB-only devices would significantly prolong the lifespan of developed applications, favoring broader adoption in daily life.

Recent innovative and high-performing Deep Learning (DL) Human Pose Estimation (HPE) frameworks are providing new momentum for RGB-only video analysis [28,29,30]. Leveraging the advantages of non-invasive video analysis, systems such as the Google MediaPipe [31] and OpenPose [32] frameworks have gained significant traction. While many applications primarily rely on estimating 2D pixel-based coordinates for tasks such as real-time Human Activity Recognition (HAR) [33], the underlying models can also extend these estimates to 3D [34] and have shown emerging utility in the analysis of movement disorders [35].

In the specific context of postural assessment, several studies have started applying HPE models. In Ref. [36], the authors used a computer vision approach on static dorsal RGB images of PD subjects to semi-automatically identify bone points for posture evaluation, showing promising classification results but highlighting sensitivity to camera-subject distance. The authors in Ref. [37] utilized key points automatically extracted by the OpenPose framework on static images to identify points of interest for post-processing algorithms, though their evaluation remained susceptible to camera positioning and rotation. The authors in Ref. [38] used the 2D pixel-based MP model to analyze postural characteristics of young adults from static images (frontal, dorsal, and lateral views) and quantify differences in angular and linear (pixel-distance) measurements between sexes. However, world-space (3D) coordinates are seldom used in these contexts; furthermore, MP’s 3D models tend to exhibit reconstruction problems under highly dynamic conditions [39].

Based on the current literature, no studies appear to have directly used the 3D models provided by Google MediaPipe for a comprehensive evaluation of postural abnormalities in PD.

To bridge this gap, the primary objective of this study is to investigate the potential and limitations of 3D skeletal models obtained from the Google MediaPipe Pose (MP) RGB-only framework for quantifying axial postural alterations in subjects with PD. This was achieved by directly comparing their performance with the reference KIN_3D model generated by the Microsoft Azure Kinect tracking algorithm. We utilized a synchronized dual-camera setup (frontal and lateral views) to capture a prolonged standing task of 40 PD patients. The choice of a 60 s task, instead of single-image analysis, allows for the detection of variations in postural alignment and balance over time during the steady task [40]. Furthermore, using cameras with recording capability allows us to robustly extract both the Azure Kinect body-tracking model and the Google MediaPipe Pose models (using only the RGB stream) from the same synchronized video recordings, facilitating direct, comparative validation. The activities were conducted as part of the OMNIA-PARK project [41], a multicenter study focused on axial symptoms in Parkinson’s disease. The project applied multi-sensor approaches (such as vision systems, surface electromyography, and wearables) combined with artificial intelligence techniques to investigate in depth postural abnormalities, gait disorders, speech alterations, and dysphagia [42] to provide a comprehensive assessment of the axial symptom effects.

The study introduces several innovative elements in the field of digital PD monitoring. First, we implement a synchronous multi-view architecture (main-frontal and sub-lateral) to overcome the inherent depth-estimation limitations of RGB-only frameworks, enabling the acquisition of angular measurements that would otherwise be inaccurate from a frontal perspective. Second, we provide a granular evaluation of MP 3D models, investigating whether increased computational complexity translates into higher measurement accuracy than RGB-D tracking. From a clinical perspective, we investigate whether objective parameters derived from these 3D markerless models effectively reflect the severity of axial PAs observed in PD patients and align with standardized clinical assessments of posture, global motor impairment, and other functional domains [43]. By addressing these questions, this study aims to define the boundaries of RGB-only human pose estimation, enhancing the development of accessible, camera-based clinical assessment tools.

## 2. Materials and Methods

### 2.1. Participants and Clinical Assessment

A total of 40 patients diagnosed with Parkinson’s Disease (PD) according to the Movement Disorder Society (MDS) clinical diagnostic criteria [44] were enrolled in this study. All participants were treated with dopaminergic therapy, and their Levodopa Equivalent Daily Dose (LEDD) was calculated using established conversion factors [45]. Participants were consecutively recruited from routine outpatient visits by expert neurologists at the Department of Neuroscience “Rita Levi Montalcini” of the University of Turin and at the Movement Disorders outpatient clinic of “Sapienza” University of Rome, following the same recruitment protocol and standardized inclusion/exclusion criteria. Inclusion was restricted to PD patients with moderate-to-advanced disease, defined as a Hoehn & Yahr (H&Y) score of 2.5–4 [46]. Exclusion criteria included: neurological signs suggestive of atypical or secondary Parkinsonism; cognitive impairment defined as a Montreal Cognitive Assessment (MoCA) score ≤ 21 [47]; inability to provide informed consent; orthopedic, rheumatologic, other neurological conditions, and/or previous surgeries significantly affecting gait or balance. The study was conducted in accordance with the Declaration of Helsinki and received ethical approval from the institutional review board (“Comitato Etico Territoriale (CET) interaziendale AOU Città della Salute e della Scienza di Torino”, Protocol 0080563 No. 512/2023, approved on 17 June 2024). All participants provided written informed consent prior to enrollment.

About 7 days before the instrumental analysis, all participants underwent a comprehensive clinical evaluation to characterize motor, cognitive, and quality-of-life domains. This assessment included: the MDS Unified Parkinson’s Disease Rating Scale (MDS-UPDRS) [48], the MoCA test, the Berg Balance Scale (BBS) [49], the Unified Dyskinesia Rating Scale (UDysRS) [50], New Freezing of Gait Questionnaire (NFOG) [51], the Parkinson’s Disease Fatigue Scale (PFS-16) [52], the King’s Parkinson’s Pain Scale (KING) [53], and the Parkinson’s Disease Questionnaire (PDQ-8) [54]. The main demographic and clinical characteristics of the participants are summarized in Table 1.

Following the clinical assessment, participants attended an instrumental acquisition session involving various motor and non-motor tasks. All clinical evaluations and instrumental acquisitions were performed while patients were on their usual dopaminergic therapy (ON state), to reflect real-life clinical conditions and ensure patient safety and compliance during tasks. The Motion Analysis laboratories of the Polito^BIO^Med Lab (Politecnico di Torino, Turin, Italy) and of the Sapienza University (Rome, Italy) hosted all the experimental sessions. The present study specifically focuses on static postural performance, consisting of two 60 s standing tasks: one with eyes open and one with eyes closed. To facilitate multi-view (and multi-sensor) data synchronization for subsequent analysis, participants were instructed to perform a single raising movement with the left arm immediately after recording began: this movement serves to temporally align the collected data for postural analysis.

### 2.2. Postural Data Acquisition System

Instrumental postural analysis was conducted using two distinct markerless human pose estimation (HPE) systems: the RGB-only Google MediaPipe Pose framework (v0.10.21) and the RGB-D skeletal tracking algorithm of Microsoft Azure Kinect (Azure Kinect Body Tracking SDK v1.1.0). This dual-system approach was designed to evaluate the consistency and reliability of an RGB-only approach compared to an RGB-D depth-based solution in quantifying PD-specific axial PAs.

A dual-camera setup featuring two Azure Kinect devices (Microsoft Inc., Redmond, WA, USA) [55] was developed for this study. To ensure temporal synchronization, the devices were connected in a daisy-chain configuration via a 3.5 mm audio cable using the dedicated *sync in* and *sync out* ports (on the back of each device). In this configuration, one camera operated as the Main and the other as the Sub, with the Sub’s recording triggered exclusively by the Main’s synchronization signal.

**Figure 1:**
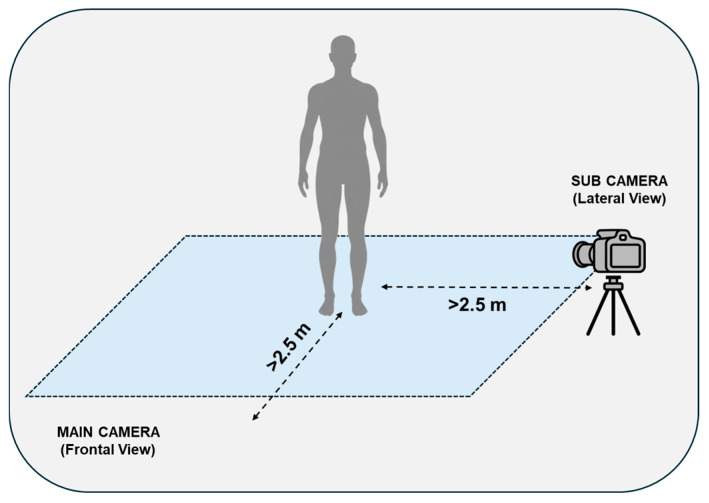
Schema of the dual-camera setup.

Both cameras were interfaced with a single mini-PC (ZOTAC^©^, Zotac, Fo Tan, New Territories, Hong Kong, China; processor: 2.4 GHz quad-core 9th generation, graphics card: NVIDIA GeForce RTX 2060 6 GB, RAM: 16 GB) via high-speed data cables (lengths: 2.5 m for Main, 5 m for Sub). The devices were mounted on tripods to ensure stability and powered externally to guarantee high performance. The Main camera was positioned directly in front of the participant, while the Sub camera was placed laterally (approximately 2.5 m) to enable simultaneous, synchronous dual-view recording of the postural tasks. The acquisition setup scheme is illustrated in Figure 1.

The recording procedure was managed through a custom-built Unity-based user interface that executed separate batch files for each device according to multi-device synchronization specifications [56]. The cameras were configured to capture RGB (1280 × 720 p, nominal field of view: 90° × 59°), Depth (Narrow Field of View unbinned mode), and Infrared (IR) streams at 30 FPS, maintaining an inter-camera delay of less than 10 msec. The raw output from each camera was a single Matroska Video (MKV) file containing the multi-modal streams for the entire duration of the task.

### 2.3. Human Pose Estimation: From Video to Skeletal Models

**Figure 2:**
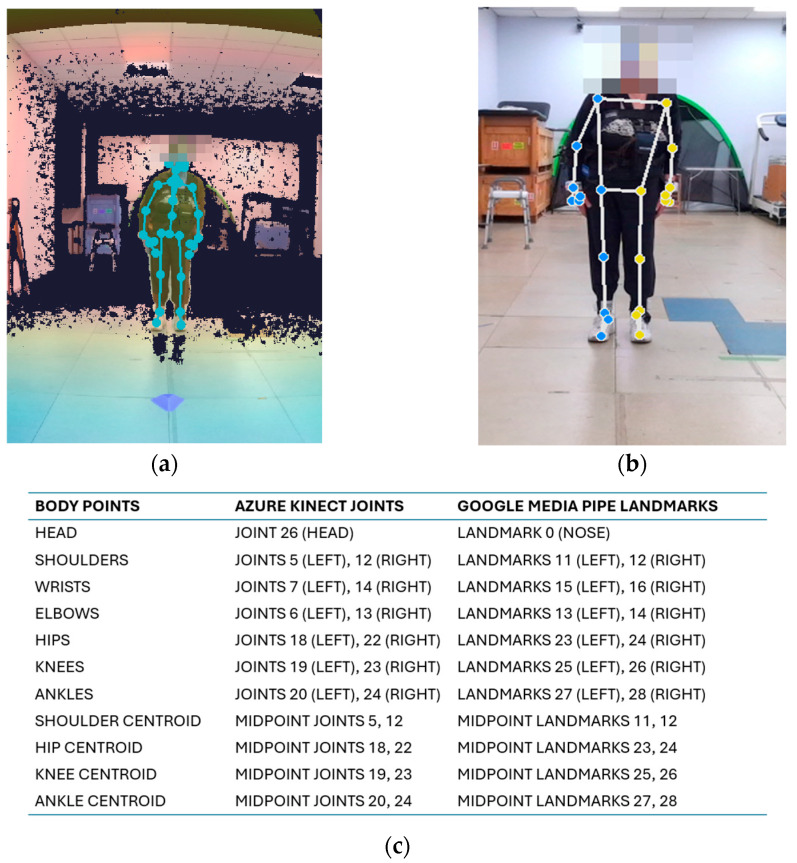
Example of complete skeletal models estimated for Azure Kinect (**a**) and Google MediaPipe (**b**) on the same subject, and (**c**) the correspondence between joints [57] and landmarks [58] considered for the postural analysis. Key points on the head are not visible due to the anonymization procedure.

The 60 s MKV recordings were processed using a custom Python 3.11.9 script to extract skeletal models from both the Google MediaPipe Pose v0.10.21 and Azure Kinect v1.1.0 frameworks. An example of skeletal model estimation is shown in Figure 2.

Google MediaPipe (MP) is an open-source single-person tracking algorithm that estimates 33 landmarks for a person detected in the camera field of view, using only the RGB stream stored in the MKV file. MP provides frame-by-frame skeletal data in 2D image coordinates and 3D world coordinates. The Python script allows configuration of model complexity (0: lite; 1: full; 2: heavy) and detection and tracking confidences (range 0–1). In this study, the confidence threshold was set to 0.7 to balance false positives (e.g., misidentifying non-human silhouettes) with consistent tracking of partially occluded landmarks. Only the 3D skeletal models were considered in the present study to enable a direct comparison with the 3D skeletal model from Azure Kinect. Three distinct MP models were generated by setting the three complexity levels; we named them MP_3D_0, MP_3D_1, and MP_3D_2, respectively. For these models, the 3D world coordinates (X, Y, Z) of each landmark are expressed relative to the model’s origin, located at the hip center.

Azure Kinect uses a proprietary multi-person tracking algorithm. It estimates the position of 33 three-dimensional joints to form a skeletal model for each detected person in the camera’s field of view (FOV). Unlike MP, the skeletal estimation is primarily based on the Depth stream of the MKV file. The resulting 3D world coordinates (X, Y, and Z) for each joint are expressed in an absolute coordinate system centered on the device. The 3D skeletal model generated by each Azure Kinect is designated KIN_3D and serves as the reference model in this study.

As shown in Figure 2, the two frameworks exhibit clear differences in the positioning of several key points: unlike KIN_3D, the MP models are simplified in the upper body and concentrate numerous landmarks on the face [57,58]. To ensure a direct comparison between the two frameworks, a specific set of common body points was identified. The mapping between joints and landmarks, including centroid calculation, is shown in Figure 2c. This set served as the basis for all subsequent angular and segment computations.

While the optimal tracking condition requires only the patient to be within the FOV (Figure 2), the varying degrees of motor impairment in the patient group necessitated the presence of at least one supporting operator for patient safety. This introduced multiple individuals in the FOV, and consequently, into the recordings, requiring specific offline procedures to isolate the patient’s skeletal data:

- For MP: As a single-person framework, MP tracks the first viable subject (not necessarily the patient) it detects. To ensure the algorithm correctly identified and locked onto the patient, a manual masking procedure was implemented in the Python script using OpenCV library. A tailored rectangular black area was defined and applied over the operator’s position during the initial frames (approximately the first second) of each RGB recorded video. This procedure obscured confounding individuals, forcing the tracker to initialize solely on the patient. Once this initial lock was established, the MP tracking successfully maintained focus on the patient for the remainder of the task, regardless of the operator’s presence.
- For KIN_3D: A custom MATLAB 2024b (MathWorks, Inc., Natick, MA, USA) procedure was developed to disambiguate the patient from the operators. The procedure used spatial filtering, prioritizing the subject positioned most centrally and closest to the camera (the patient), ultimately discarding all other, more lateral and farther-away detected skeletons (the operators). In cases where the operator was necessarily too close to the patient to allow reliable spatial separation of tracked skeletons, as occurred for one additional patient not included in this study, the recordings were discarded to ensure the integrity of the reference data.

The final output of this phase consists of MAT files for each perspective: for both Main and Sub views, one file was generated for the KIN_3D reference and three for the MP variants, providing directly usable information for the subsequent analysis.

### 2.4. Trajectories Preprocessing

The 3D trajectories of all skeletal points (joints for KIN_3D and landmarks for MP) were processed and analyzed using custom-developed MATLAB scripts (version 2024b). A standardized preprocessing pipeline was applied to the raw 3D data (i.e., trajectories) from both views and across all skeletal models (KIN_3D, MP_3D_0, MP_3D_1, and MP_3D_2). The preprocessing procedure consists of the following sequential steps:

- Resampling: All data were resampled at 50 Hz to remove timestamp jitters, ensure a uniform temporal baseline, and increase the sample density.
- Median filtering: A 10-sample median filter was applied to each trajectory component (X, Y, and Z) to remove sporadic spikes or outliers.
- Low-pass filtering: High-frequency noise was mitigated using a low-pass Butterworth filter (4th-order, 5 Hz cut-off frequency), ensuring that the resulting signals retained only the frequencies relevant to static postural analysis.

The choice of resampling and filtering frequencies is supported by evidence that postural sway dynamics in PD are primarily characterized by low-frequency components, with physiologically relevant information occurring well below 5 Hz [59,60].

Subsequently, the preprocessed trajectory of the left wrist was used to identify a stable 40 s window for the postural analysis. The temporal window was defined starting from T_I_ (3 s after the peak of left wrist raising) to T_F_ (40 s after T_I_). This window selection was designed to isolate the period of maximum postural stability, minimizing potential artifacts from incidental body movements or adjustments typically occurring at the beginning and end of the recording session. All joint and landmark trajectories were trimmed to this specific 40 s window for the estimation of postural parameters.

### 2.5. Estimation of Postural Parameters

The postural analysis was restricted to body points (joints and landmarks) common to all models (KIN_3D and MP): the head, shoulders, hips, knees, and ankles. Since the MP framework does not estimate a dedicated landmark for the head center, the Nose landmark was used as a proxy for head position in all corresponding MP angular measurements. This choice was based on its stable, central position and its superior visibility compared to other facial landmarks, thus ensuring robust tracking even in the presence of minor head movements. A custom MATLAB script was developed to estimate postural parameters from the preprocessed 3D trajectories, enabling a comprehensive assessment of posture through four groups of angular measurements.

Horizontal parameters (frontal plane, Main view) evaluate the alignment of bilateral joints relative to the horizontal line. They were computed as the angle between the segment connecting bilateral body points and the *X*-axis in the frontal plane (XY), excluding the Z component. This group includes the horizontal alignment of the shoulders (H_M__SHOULD), hips (H_M__HIP), knees (H_M__KNEE), and ankles (H_M__ANKLE).

Vertical parameters (frontal plane, Main view) evaluate the lateral body bending by computing the angle between a body segment and the *Y*-axis (vertical) in the frontal plane (XY), excluding the Z component. This group includes the alignment of the upper trunk (V_M__TRUNK, defined between the shoulder and hip centroids), the head (V_M__HEAD, between the head and the shoulder centroid), and the lower body (V_M__ANKLE, between the hip and ankle centroids).

Sagittal parameters (Main and Sub views) evaluate the anteroposterior (forward-backward) lean of body segments. In this context, the term “sagittal” refers to the anatomical plane of the postural measurements, while “frontal” and “lateral” denote the camera perspective (Main and Sub views, respectively). These angles are defined as the angle between a body segment and the *Z*-axis in the sagittal plane YZ for the Main view, and as the angle between a body segment and the *X*-axis in the frontal plane XY for the Sub view. For the frontal view (Main camera), this group includes the bending for the upper trunk (Z_M__TRUNK, segment between shoulder and hip centroids), head (Z_M__HEAD, segment between head and shoulder centroid), knee (Z_M__KNEE, segment between hip and knee centroids), and ankle (Z_M__ANKLE, segment between knee and ankle centroids). For the lateral view (Sub camera), the same angles are computed using only the left-side joints, which were the most visible and consistently tracked from the lateral perspective. Specifically, angles for the upper trunk (Z_S__TRUNK, segment between left shoulder and left hip), head (Z_S__HEAD, segment between head and left shoulder), knee (Z_S__KNEE, segment between left hip and left knee), and ankle (Z_S__ANKLE, segment between left knee and left ankle) were estimated.

Joint 3D angles (Main and Sub views) evaluate joint-specific 3D angles. For the frontal view, angles were computed separately for both left (L) and right (R) sides to detect potential asymmetries in the shoulders (L_M__SHOULD and R_M__SHOULD), hips (L_M__HIP and R_M__HIP), and knees (L_M__KNEE and R_M__KNEE). For the lateral view, angles for the elbow (L_S__ELB), hip (L_S__HIP), and knee (L_S__KNEE) were estimated for the left-body side.

**Figure 3:**
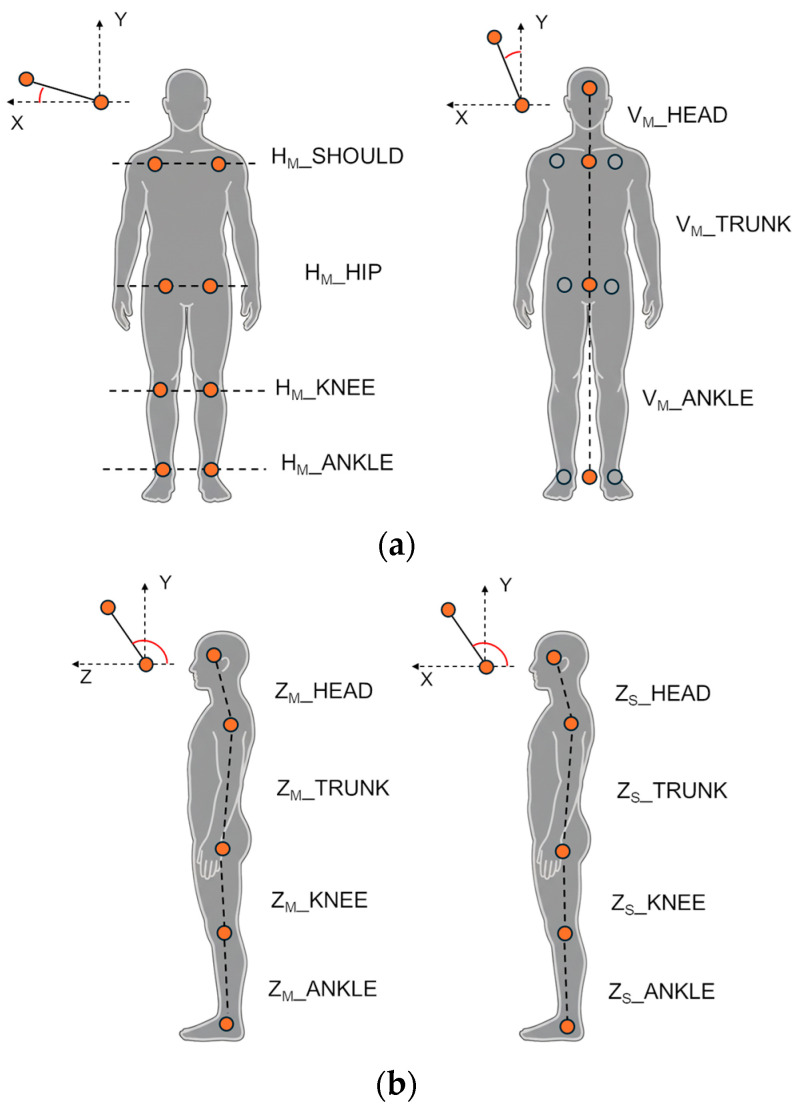

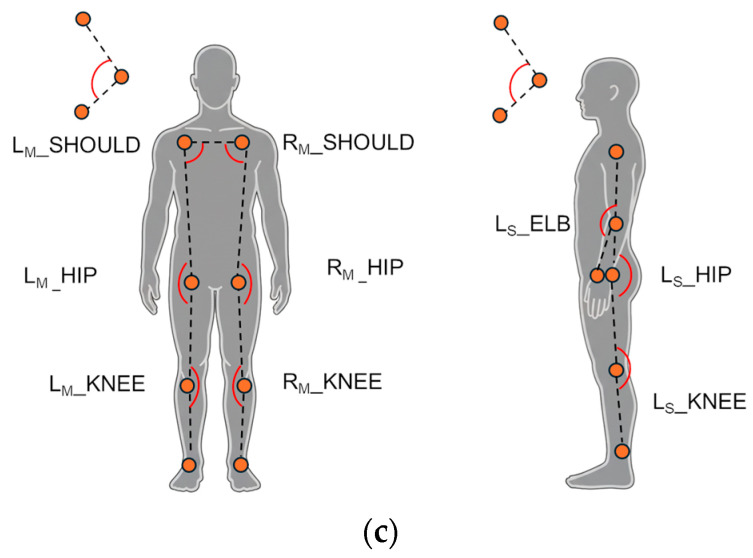
Angles computed for the analysis: Horizontal and vertical angles from the frontal (Main) view (**a**); sagittal angles from the frontal (Main) and lateral (Sub) views (**b**); joint 3D angles from the frontal (Main) and lateral (Sub) views (**c**). Orange circles indicate the approximate positions of anatomical body points, while dashed black lines represent the body segments used for angle estimation.

Figure 3 illustrates the estimated angles for frontal and lateral views.

In addition, segment lengths (Main view) were computed to measure the length of key body segments. In particular, the shoulder width (W_M__SHOULD) and hip width (W_M__HIP) were computed to highlight differences between KIN_3D and MP models at crucial body points involved in angular measurement.

Angular measures between two segments (vectors *v*_1_ and *v*_2_) were computed using Equation (1):

(1) $$\theta =atan2\;\left(\|v_{1}\times \;v_{2}\|,v_{1}\cdot v_{2}\right)$$

Segment lengths were calculated as the Euclidean distance between two 3D points (*P*_1_ and *P*_2_), as shown in Equation (2).

(2) $$d\left(P_{1},P_{2}\right)=\sqrt{{\left(x_{2}-x_{1}\right)}^{2}+{\left(y_{2}-y_{1}\right)}^{2}+{\left(z_{2}-z_{1}\right)}^{2}}$$

Finally, a Symmetry Index (SI) was computed to assess differences between the left (L) and right (R) sides of the body using Equation (3).

(3) $$SI=1-\left(\left|R-L\right|\right)/\left(\left|R\right|+\left|L\right|+\varepsilon \right)\;\;\;\;\;\varepsilon \approx \;10^{-6}$$

where ε is a small constant added to prevent division by 0. SI values close to 1 indicate near-perfect symmetry, while values near 0 indicate maximum asymmetry.

### 2.6. Statistical Analysis

For the statistical analysis, data from both eyes-open and eyes-closed conditions were pooled into a single dataset. This approach was adopted to evaluate the models’ performance across a larger dataset (80 recordings: 40 with eyes open and 40 with eyes closed), consistent with the study’s primary objective of validating measurement consistency rather than assessing sensory-related differences.

The KIN_3D and MP 3D models were initially compared by calculating mean and standard deviation (SD) for all estimated angles to summarize the central tendency of postural alignment and instability across the cohort. To assess the statistical significance of differences in angular measurements between the three MP variants and the KIN_3D model, the Wilcoxon Signed-Rank Test was used. This non-parametric choice was selected due to the paired nature of the data and to ensure robustness against potential non-normality in the differences between measurements, as suggested by the Shapiro–Wilk normality test [61].

The statistical relationship and consistency between models were evaluated using Pearson’s correlation coefficient; this choice is particularly suited to assessing the proportionality between instrumental systems across continuous measurements. This analysis was conducted at two levels: inter-model consistency, evaluating the relationship between each MP variant and the reference KIN_3D model; and intra-model consistency, assessing the relationship between pairs of MP models to estimate the impact of algorithmic complexity on measurement performance.

For joint angles (Main view only), the Symmetry Index was compared across all models to evaluate postural asymmetries and investigate whether the different tracking algorithms influenced the detection of bilateral discrepancies. Furthermore, Pearson’s correlation was used to assess the clinical validity of a subset of objective parameters by examining the strength and significance of their association with the standardized clinical scales and scores. To complement the analysis, the Mean Absolute Error (MAE) and Bland–Altman metrics (systematic bias and 95% Limits of Agreement) were calculated to quantify the discrepancy between the MP and the reference models for the selected subset.

A more in-depth investigation was finally performed based on the clinical assessment of posture (MDS-UPDRS item 3.13): the cohort was divided into two clusters according to the assigned severity score. In addition, Principal Component Analysis (PCA) was performed to investigate the relationship between postural parameters and clinical severity. Prior to PCA, all variables were standardized using Z-score normalization to ensure equal weighting. The PCA was computed independently for each model (KIN_3D, MP_3D_0, and MP_3D_1) to assess their respective capacities to represent clinical stratification. The first three principal components (PC1, PC2, and PC3) were retained for 3D visualization, and 95% confidence ellipsoids were calculated for each severity group (Cluster 1 vs. Cluster 2). Finally, the feature weights on the first principal component (PC1) were examined to rank the contribution of specific postural angles to the overall variance, providing an objective measure of feature importance for clinical stratification.

All statistical computations and PCA visualizations were performed using MATLAB (version R2024b). Statistical significance for all tests was set at *p*-value < 0.05.

## 3. Results

### 3.1. Angular Measurement Analysis Across 3D Markerless Models

In this section, a comparative analysis of markerless models is conducted to assess the reliability and consistency of angular measurements.

#### 3.1.1. Horizontal Angles (Main View)

Table 2 presents the mean and standard deviation for the horizontal alignment angles obtained from the patient cohort. In addition, the statistical significance of the differences in angular measurements between each MP model and KIN_3D is reported.

The MP_3D_0 and MP_3D_1 models exhibited high congruence with the KIN_3D reference for upper-body alignment (shoulders and hips), as indicated by both mean values and standard deviations, without significant differences. Conversely, a significant discrepancy was observed for lower-body measurements (knees and ankles), which increased with model complexity. Specifically, the MP_3D_2 model introduced substantial errors, particularly in the shoulders (12.19° vs. 4.22° for KIN_3D) and the knees (26.45° vs. 2.81° for KIN_3D), while remaining congruent only in hip alignment. This behavior, indicative of a distorted 3D reconstruction due to erroneous depth estimation, is consistent with previous observations in both dynamic [39] and static conditions in healthy populations [62].

Correlation analysis (Table 3) further supports these findings. For horizontal angles, a significant moderate-to-strong correlation (ρ = 0.57–0.72) was found between the lower-complexity MP models (MP_3D_0 and MP_3D_1) and KIN_3D for shoulder and hip alignment. Poor correlation was observed in the lower limbs. The correlation with KIN_3D for knee and ankle measurements was negligible for MP_3D_0 and weak for MP_3D_1. Although the MP_3D_2 maintained a moderately significant correlation for shoulders and hips (ρ ≥ 0.49), the substantial absolute bias in mean angles (Figures in S1 of Supplementary Materials) indicates that this model applies a systematic offset across the cohort, rather than failing to capture inter-subject variability, a phenomenon previously noted in healthy subjects [62].

Regarding internal consistency, the three MP variants demonstrated strong reciprocal correlations for hip alignment and moderate correlations for the shoulders. Notably, MP_3D_1 and MP_3D_2 models remained moderately-to-strongly correlated across all angular measures (ρ ≥ 0.61), suggesting they share a common algorithmic foundation for variability estimation, despite the significant reconstruction errors in the highest complexity model (MP_3D_2).

In summary, MP frameworks demonstrate promising reliability and strong correlation with RGB-D (KIN_3D) measurements for the horizontal alignment of the hips and shoulders estimated from a frontal view, except for MP_3D_2, which shows a high systematic bias. Reliability significantly degrades for the lower limbs (knees and ankles). Consequently, the use of MP models for horizontal alignment is comparable to RGB-D sensors only for upper-body assessment and should be restricted to complexities 0 and 1.

#### 3.1.2. Vertical Angles (Main View)

Table 4 presents the mean and standard deviation values for the vertical (lateral flexion) angles obtained from the reference sample. In addition, the statistical significance of the differences in angular measurements between each MP model and KIN_3D is reported.

All three MP models exhibited high congruence with the KIN_3D reference in measuring lateral body flexion. A slight discrepancy was noted in the mean V_M__HEAD angle (alignment of the head relative to the shoulder center) for lower complexities (MP_3D_0 and MP_3D_1), which appeared to converge toward the reference value in the MP_3D_2 model. The high absolute mean head angle is consistent with the clinical profile of the cohort, which includes subjects with Camptocormia and Pisa Syndrome, in which the head assumes an anomalous lateral alignment relative to the shoulder center. Figures in S2 of the Supplementary Materials delves into the observed behavior.

These findings were further supported by Pearson’s correlation analysis (Table 5). The results demonstrated a strong correlation (ρ ≥ 0.79) between all MP models and KIN_3D in measuring lateral trunk flexion (V_M__TRUNK). However, the correlation for V_M__HEAD was only moderate for MP_3D_0 and MP_3D_1 (ρ < 0.50), reflecting a systematic underestimation bias. Crucially, the MP_3D_2 model achieved the highest correlation (ρ = 0.76) with KIN_3D for the head angle, indicating superior performance for this specific anatomical metric in a pathological context. This result partially contradicts previous observations in healthy subjects [62], where the heavy model showed significant errors. It is likely that the pronounced postural deviation in PD patients (e.g., severe lateral leaning) provides clearer visual features, enabling the more complex MP model to resolve measurement challenges more effectively.

Conversely, the distal lower-body measurement (V_M__ANKLE) showed negligible correlations with KIN_3D across all MP models. This lack of correlation likely reflects the restricted range of observed values and the minimal differences in mean and standard deviation compared to KIN_3D. In such cases, even subtle measurement noise or slight discrepancies can affect the correlation coefficient, despite the small absolute error.

#### 3.1.3. Sagittal Angles (Main and Sub Views)

Table 6 presents the mean and standard deviation for the sagittal angles obtained from the reference sample. The statistical significance of the differences in angular measurements between each MP model and KIN_3D is also reported.

In the frontal perspective (Main view), all MP models consistently overestimated the sagittal angles of the trunk and ankles, exhibiting a significant absolute bias (e.g., Z_M__TRUNK is around 109.55° for MP vs. 99.93° for KIN_3D). While the knee angle maintained slightly more coherence with the reference model (KIN_3D), a difference of several degrees persisted. This systematic overestimation likely stems from a fundamental divergence in landmark localization (MP) and joint positioning (KIN_3D).

Regarding the head angle (Z_M__HEAD), MP measurements were excessively high, with the error significantly greater in MP_3D_2 (mean: 159.44°). This behavior is attributable to the inherent challenge of estimating depth from a single frontal RGB stream. Crucially, this effect was significantly mitigated in the lateral perspective (Sub view), where MP measurements were generally more closely aligned with KIN_3D, although a substantial offset in Z_S__HEAD persisted due to differing anatomical reference points (Figure 2), especially between the head joint (KIN_3D) and the head point estimated from face landmarks (MP). It is important to note that, unlike the frontal perspective, angular measurements from the Sub camera were derived only from the left side of the body, which is more visible in the implemented setup. Figures in S3 of the Supplementary Materials delves into the observed behavior.

Pearson’s correlation analysis (Table 7) highlights the superior performance of the lateral perspective compared to the frontal view for antero-posterior assessment. This result strongly supports the advantage of the multi-camera system implemented in this study. Specifically, correlations with KIN_3D markedly improved in the lateral view for the trunk (ρ ≥ 0.87), knee (ρ ≥ 0.72 for MP_3D_0 and MP_3D_1), and ankle (ρ = 0.65 for MP_3D_2). The notable exception was the head angle (Z_S__HEAD), which showed a lower correlation than in the frontal view, likely because the Nose landmark is noisier when viewed in profile. Conversely, internal consistency (intra-model correlation) among MP variants showed conflicting results regarding camera positioning. While congruency improved for the trunk and knee angles from the lateral perspective, a significant loss of consistency was observed for the ankles across all models and for the head at higher complexities.

In conclusion, while MP models exhibit systematic absolute bias (e.g., approximately 10° overestimation for Z_M__TRUNK), they show moderate-to-strong correlations with KIN_3D in quantifying trunk flexion severity from both perspectives. Furthermore, the use of the lateral view (Sub camera) significantly enhanced coherence and correlation for lower-limb angles (Z_S__KNEE, Z_S__ANKLE), proving advantages for reliable antero-posterior postural analysis.

#### 3.1.4. Joint Angles (Main and Sub Views)

Table 8 presents the mean and standard deviation values for the 3D joint angles obtained from the reference sample. The statistical significance of the differences in angular measurements between each MP model and KIN_3D is also reported.

In the frontal perspective (Main View), all models show congruence in shoulder angle measurements and consistently highlight a clear discrepancy between the right and left sides of the body. This discrepancy is attributable to actual postural asymmetry within the cohort, where several subjects exhibit marked lateral trunk flexion that misaligns the shoulder line. The MP models, particularly MP_3D_0 and MP_3D_1, show high absolute coherence with KIN_3D in capturing this asymmetry, despite a minimal systematic offset. Conversely, the MP_3D_2 model exhibits a substantial asymmetry at the hips, a discrepancy not observed in the other models, which is likely linked to distortions in the 3D skeletal reconstruction, as previously reported for this complexity [62].

Furthermore, all MP models underestimate knee angles by approximately 10–12°. This bias, coupled with the poor correlation in the frontal perspective (Table 9), is largely explained by the fundamental difference in the reference-point definitions between KIN_3D (joints) and MP (landmarks) models (Figure 2). This hypothesis is strongly supported by the analysis of segment lengths. Specifically, shoulder and hip widths were measured as 0.31 ± 0.01 m and 0.16 ± 0.01 m for KIN_3D; 0.33 ± 0.01 m and 0.21 ± 0.01 m for MP_3D_0; 0.34 ± 0.01 m and 0.23 ± 0.01 m for MP_3D_1; and 0.33 ± 0.01 m and 0.21 ± 0.01 m for MP_3D_2. This dimensional discrepancy confirms that the angular estimations are derived from different anatomical reference points.

For the lateral perspective (Sub View), the MP_3D_2 model exhibited clear inaccuracies, overestimating the left elbow and hip angles while underestimating the knee angle. In contrast, MP_3D_0 and MP_3D_1 appeared more coherent with KIN_3D. Notably, KIN_3D showed excessive elbow-angle variability (high standard deviation), likely due to the inherent difficulty of the Azure Kinect tracking algorithm in stably estimating wrist joint position from a lateral profile. Despite this, Table 9 shows stronger correlations for angles estimated from the lateral perspective, consistent with previous findings and further supporting the utility of a dual-perspective setup. Figures in S4 of the Supplementary Materials delves into the observed behavior.

**Figure 4:**
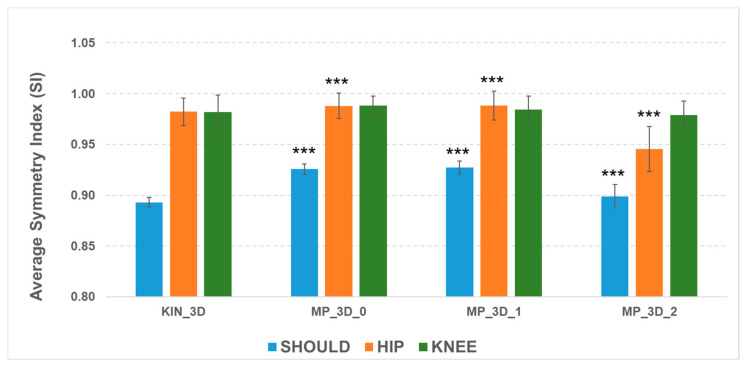
Average Symmetry Index (SI) across angles and models. Symbols indicate statistical significance of MP models vs. KIN_3D (***: *p* < 0.001).

The ability of the MP frameworks to reflect relevant asymmetry, despite absolute measurement biases, was examined via the Symmetry Index (SI) for bilateral joints (Figure 4).

The SI analysis shows that all models effectively detect shoulder misalignment, as evidenced by lower SI values, though the MP models differ statistically from KIN_3D due to biases in angular measurements. For the hips, all models maintained high symmetry (SI > 0.98), except for MP_3D_2 (SI = 0.94), reinforcing the evidence of reconstruction issues in the high-complexity model. Knee SI values were similar across all models, with a slight reduction for MP_3D_2. These results confirm that MP_3D_0 and MP_3D_1 models can reliably estimate bilateral symmetry, making them suitable for detecting also postural asymmetries in Parkinson’s Disease, whereas MP_3D_2 should be avoided due to significant hip-level distortions.

#### 3.1.5. Summary of Technical Comparison and Selection of Parameters

The technical validation, encompassing horizontal, vertical, sagittal, and 3D joint angles, enabled a robust identification of the most reliable MP models and measurement perspectives for the subsequent clinical validation. In interpreting the main findings, it should be noted that while KIN_3D serves as the reference model in this study, it is not a gold standard. Residual tracking instabilities inherent to Azure Kinect, particularly regarding distal joints, may propagate into reference measurements, potentially contributing to the observed discrepancies. Consequently, the reported results reflect a relative comparison between markerless models. A more in-depth validation of KIN_3D versus a marker-based gold standard is available in our previous work [62].

To move towards clinical validation, we selected a subset of postural parameters that can be reliably estimated by markerless models and that capture inter-subject variability, as confirmed by high, statistically significant Pearson correlation coefficients. Based on these criteria, the following parameters were selected:

- Horizontal Angles (Main View): H_M__SHOULD and H_M__HIP
- Vertical Angles (Main View): V_M__TRUNK and V_M__HEAD
- Sagittal Angles (Main View): Z_M__TRUNK and Z_M__HEAD
- Sagittal Angles (Sub View): Z_S__TRUNK, Z_S__HEAD, and Z_S__KNEE
- Joint Angles (Main View): L_M__HIP and R_M__HIP
- Joint Angles (Sub View): L_S__ELB, L_S__HIP, and L_S__KNEE

As emerged from the previous analyses, the lower-complexity MP models (MP_3D_0 and MP_3D_1) show the highest coherence with KIN_3D, exhibit lower systematic offsets, and better preserve bilateral symmetry. To objectively assess the systematic offsets of the different MP models relative to KIN_3D, we calculated the Mean Absolute Error (MAE) and Bland–Altman metrics (bias and Limits of Agreement) for the selected parameters (Table 10).

The metrics in Table 10 confirm the distinct performance profiles among the MP models previously evidenced. MP_3D_0 and MP_3D_1 exhibit lower systematic bias and high precision for trunk and hip metrics (MAE < 2°). Other parameters exhibit larger absolute discrepancies with KIN_3D. These discrepancies are primarily attributable to the dissimilar skeletal configurations used by the models. In fact, the different positioning of joints and landmarks results in a skeletal structure with a different underlying geometry, thereby generating different angles between body segments. This is reflected in Bland–Altman and MAE metrics. However, the high correlation coefficients reported in previous sections confirm that these offsets are systematic, meaning the MP models remain sensitive to inter-subject variations and clinical postural trends.

In contrast, MP_3D_2 exhibited critical failures arising from a distorted 3D reconstruction from a single camera view, as observed in healthy subjects [62]. This reconstruction introduced severe biases and significant loss of skeletal symmetry, as evidenced by the high errors in straightforwardly estimated parameters, such as H_M__SHOULD, L_M__HIP, and R_M__HIP.

Consequently, MP_3D_2 was excluded from clinical analysis. The final comparison was thus restricted to MP_3D_0, MP_3D_1, and KIN_3D to evaluate the capabilities of RGB-only models to characterize pathological posture as the RGB-D reference does, despite the inherent bias introduced by skeletal mismatch.

### 3.2. Markerless Models and Clinical Assessment

This section evaluates the clinical validity of the selected markerless models by comparing objective measurements with the clinical assessments performed on the reference sample (40 subjects with PD). As previously mentioned, the clinical protocol included multiple validated, standardized scales covering motor symptom severity, balance and gait impairment, motor complications (presence and severity), non-motor symptom severity, and PD-related impairment in activities of daily living. Although several scales are not exclusively focused on posture, they were included in this analysis to explore whether static postural alignment reflects broader motor and functional domains.

**Figure 5:**
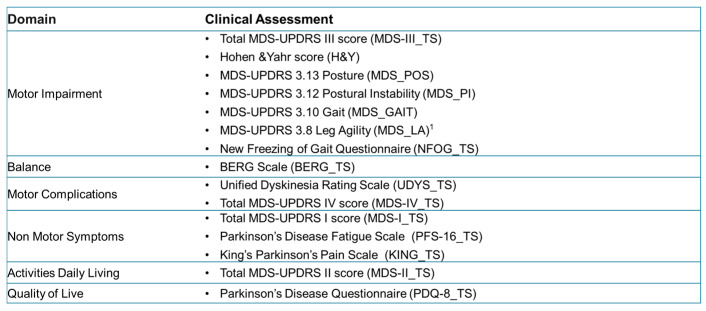
Clinical scales and scores assigned by neurologists. ^1^: Mean value of left and right Leg Agility assessment.

**Figure 6:**
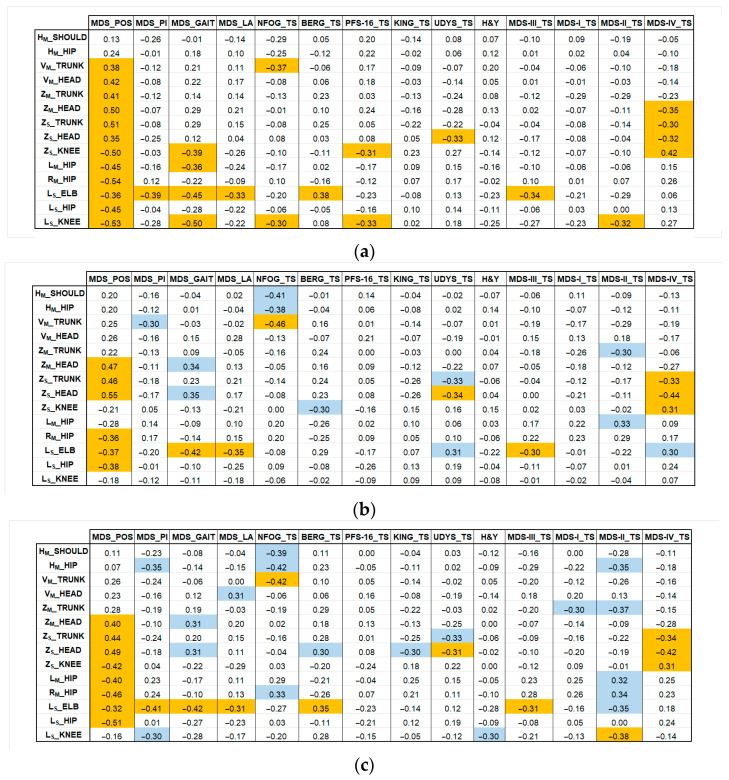
Correlations between angular parameters and clinical scales/scores for KIN_3D (**a**), MP_3D_0 (**b**), and MP_3D_1 (**c**). Orange cells indicate correlations with |ρ| > 0.3 for KIN_3D and those shared by MP_3D_0 (**b**) and MP_3D_1 (**c**). Orange cells highlight correlations with |ρ| > 0.30 unique shared by all models. Blue cells highlight additional correlations with |ρ| > 0.30 unique to the MP models.

The following scores, assigned by a team of neurologists, were categorized by clinical domain (Figure 5):

The final stage of the models’ validation was to assess the clinical relevance of the selected postural parameters by calculating the Pearson correlation coefficients (ρ) between the parameters (from KIN_3D, MP_3D_0, and MP_3D_1) and these established clinical scales and scores. Figure 6 illustrates the correlation coefficients for KIN_3D (a), MP_3D_0 (b), and MP_3D_1 (c), allowing for a direct comparison of the three models. In Figure 6a, correlations with |ρ| > 0.30 for KIN_3D between angular parameters and clinical scores for KIN_3D are displayed in orange. In Figure 6b,c, orange cells indicate correlations shared by both MP_3D_0 and MP_3D_1 models, highlighting the most consistent associations with clinical scales across all models. Conversely, blue cells indicate correlations with |ρ| > 0.3 unique to the MP models, identifying areas where RGB-only estimation might provide alternative or specific clinical insights.

#### 3.2.1. Motor Impairment and Balance

The correlation analysis between the selected postural metrics and clinical scales evaluating motor impairment (MDS_POS, MDS_GAIT, MDS_LA, MDS-III_TS, H&Y, and NFOG_TS) and balance (MDS_PI, BERG_TS) reveals a distinct hierarchy of predictive capabilities, strongly dependent on the measurement plane and the specific joint under investigation.

For KIN_3D, almost all selected parameters, except for horizontal shoulder and hip alignments, show a significant relationship with the clinical posture assessment (MDS_POS). This demonstrates that both frontal and lateral view parameters successfully capture the complexity of axial PAs in PD. The hip parameters (L_M__HIP and R_M__HIP) further quantify the observed frontal postural asymmetry. The MP models align closely with this behavior, exhibiting slightly lower but significant correlations in the same direction, thereby confirming their capability to quantify axial PAs from both frontal and lateral perspectives.

The MP models differ significantly from KIN_3D in the lateral view knee angle (L_S__KNEE), a joint crucial for comprehensive clinical assessment. Specifically, the knee parameters (L_S__KNEE and Z_S__KNEE) show a strong inverse correlation with the gait item (MDS_GAIT) for KIN_3D, meaning a more flexed knee angle correlates with greater gait impairment. This clinically relevant relationship is not consistently replicated by the MP models. This finding reinforces the previously noted performance limitations of the RGB-only framework compared to the RGB-D model in accurately reconstructing distal lower-limb parameters.

Conversely, all models show consensus regarding the elbow angle (L_S__ELB), which exhibits a consistent inverse correlation with gait (MDS_GAIT), postural instability (MDS_PI), and leg agility (MDS_LA) items.

Considering overall motor impairment (MDS-III_TS), only the L_S__ELB parameter shows a consistent inverse correlation across all models: a smaller angle (indicating a more flexed arm) is associated with higher overall severity scores. The same pattern holds true for balance (BERG_TS), where increased elbow flexion appears as a marker of a more significant balance disorder.

Other postural parameters show weak, non-significant correlations with MDS-UPDRS_III_TS and BERG_TS, scales that assess diverse functions not exclusively restricted to static posture. Similarly, correlations with the H&Y stages are weak, indicating that the overall stage of disease progression reflects cumulative disability that is not strictly predicted by static postural alignment alone, but rather by a combination of dynamic and functional factors.

Interestingly, a significant negative correlation was observed across all markerless models between metrics of trunk, shoulders, and hips (V_M__TRUNK, H_M__SHOULD, H_M__HIP) and the severity of freezing of gait (NFOG_TS). The MP models appeared to amplify this relationship in specific parameters (e.g., V_M__TRUNK in MP_3D_1: ρ = −0.46). Interpreting this result requires caution, as minor angular values in the horizontal and vertical directions (i.e., apparently better posture) are associated with higher freezing-of-gait scores. Freezing of gait is a dynamic phenomenon, primarily related to deficits in anticipatory postural adjustments, axial rotation, and gait initiation, rather than to static postural alignment during quiet standing. The observed inverse correlations, therefore, effectively reflect a dissociation between static posture and dynamic locomotor control, rather than a causal relationship.

#### 3.2.2. Motor Complications

Postural metrics demonstrated the strongest clinical relevance in predicting motor complications (MDS-IV_TS) and dyskinesias (UDYS_TS).

The three models were highly consistent in identifying a correlation between a specific subgroup of sagittal parameters derived from the lateral view (Z_S__TRUNK, Z_S__HEAD, Z_S__KNEE) and the overall motor complications scores (MDS-IV_TS), showing comparable correlation strengths across all models.

The sagittal head angle from lateral view (Z_S__HEAD) emerged as a consistently sensitive predictor of complication severity. KIN_3D showed a significant negative correlation (ρ = −0.32) with MDS-IV_TS, which was not only replicated but strengthened by the MP models (both MP_3D_0 and MP_3D_1 showed ρ ≥ −0.42). A similarly robust negative correlation was found between Z_S__HEAD and UDYS_TS.

At first glance, this inverse relationship may appear counterintuitive, as higher motor complication scores are associated with smaller sagittal deviations (i.e., apparently better head alignment). However, this pattern reflects a well-known clinical dissociation between rigid axial and hyperkinetic phenotypes in advanced PD.

MDS-IV_TS and UDYS_TS capture the global burden of motor complications, encompassing both fixed postural deformities and dynamic dyskinetic movements. In patients with a predominantly rigid-axial phenotype, severe motor complications are frequently accompanied by marked postural abnormalities, possibly also at the neck in the form of antecollis, resulting in a persistent pathological Z_S__HEAD angle.

Conversely, patients with severe generalized dyskinesias may exhibit increased trunk and neck variability even during quiet standing. Although their posture is functionally unstable, this variability reduces the mean sagittal deviation measured over a static acquisition, leading to apparently less altered Z_S__HEAD values.

Importantly, the angular metrics used in this study quantify the predominant static alignment and do not capture postural variability. As a result, Z_S__HEAD preferentially reflects the burden of fixed axial rigidity rather than the amplitude of involuntary movements.

Within this framework, the observed negative correlation indicates that higher complication scores are associated with a shift toward rigid axial involvement, which dominates the static postural signature captured by both RGB-D and RGB-only systems.

Interestingly, the MP models also identified a significant correlation for the trunk flexion parameter (Z_S__TRUNK), a relationship not detected by KIN_3D. This suggests that the RGB-only framework may offer unique sensitivity in linking trunk rigidity and flexion to the functional impact of motor complications.

#### 3.2.3. Non-Motor Symptoms

The correlation analysis between postural parameters and clinical scales evaluating Non-Motor Symptoms (NMS) revealed interesting relationships with perceived fatigue (PFS-16_TS).

Unlike the MP models, which showed only weak correlations, the KIN_3D model detected a significant negative correlation between the knee angles Z_S__KNEE (ρ = −0.31) and L_S__KNEE (ρ = −0.33) and the perceived fatigue scale (PFS-16_TS). This inverse relationship indicates that a more flexed knee (i.e., reduced angular extension) is associated with higher perceived fatigue. This may reflect the increased muscular cost of maintaining a crouched posture, leading to earlier exhaustion and a greater subjective sense of fatigue.

In contrast, the markerless models consistently indicate the absence of significant correlations (|ρ| < 0.30) between almost all postural parameters and the Pain Scale (KING_TS) and MDS-UPDRS Section I (MDS-I_TS). This suggests that static postural alignment, while closely linked to motor complications and specific functional impairments, is not a robust predictor of pain or other non-motor experiences, such as mood or sleep disorders, in this patient cohort.

#### 3.2.4. Activities of Daily Living

In general, the postural parameters demonstrated weak correlation with the assessment of Activities of Daily Living (MDS-II_TS) across both KIN_3D and MP_3D_0 models. Conversely, the MP_3D_1 model exhibited significant correlations with this score for specific parameters, primarily those derived from the frontal (Main) view, such as H_M__HIP, Z_M__TRUNK, L_M__HIP, and R_M__HIP. From the lateral (Sub) view, the knee angle (L_S__KNEE) was the notable exception, showing a significant correlation across both KIN_3D and MP_3D_1.

The observed correlation between an apparently more upright posture (indicated by smaller H_M__HIP and Z_M__TRUNK deviations, or higher L_M__HIP and R_M__HIP extension) and higher MDS-II_TS scores indicating worse ADL performance, which could possibly be related to a different phenotype of patients regarding trunk rigidity, highlights a dissociation between static postural alignment and functional performance in daily life. Patients who appear more aligned during quiet standing may still experience substantial disability due to bradykinesia, rigidity, impaired balance, and difficulty in postural transitions, features that are central to MDS-II but not captured by static posture metrics.

The MP_3D_1 model appears to be more sensitive to this compensated, rigid posture rather than a genuinely functional and flexible one. However, such static alignment does not equate to functional efficiency and may coexist with significant limitations in mobility and autonomy. Overall, these findings reinforce the concept that static postural assessment provides complementary (but not substitutive) information regarding functional disability in PD, and that preserved visual alignment should not be assumed to indicate better performance in activities of daily living.

#### 3.2.5. Relationship with Postural Severity

To evaluate the clinical relevance of the objective measurements, participants were divided into two groups based on the MDS-UPDRS item 3.13 (Posture) score: Cluster 1 (*n* = 30 samples; including patients with scores 0–1, representing no or mild alteration) and Cluster 2 (n = 50 samples; including patients with scores 2–4, representing evident axial PAs). Specifically, the cohort distribution across the clinical assessment was: 4 patients with score 0, 11 with score 1, 11 with score 2, 12 with score 3, and 2 with score 4. Table 11 summarizes the angular measurements (mean and standard deviations) for these clusters across the validated models.

All models demonstrated a consistent ability to discriminate between the two severity clusters. Although statistically significant differences were observed only for a limited subset of parameters, the trends were stable across models.

Specifically, horizontal angles (H_M__SHOULD and H_M__HIP) identified greater joint misalignment in Cluster 2, correctly intercepting the increased asymmetries associated with advanced disease stages. The hip misalignment was more pronounced in KIN_3D than in the MP models; this discrepancy likely arises from the MP reconstruction process, in which the hip center serves as the coordinate system origin, potentially reducing absolute angular deviations.

Lateral flexion (V_M__TRUNK and V_M__HEAD) and sagittal lean metrics (Z_M__TRUNK, Z_M__HEAD, Z_S__TRUNK, and Z_S__HEAD) clearly differentiated the two clusters. Cluster 2 exhibited significantly higher forward and lateral flexion, consistent with postural alterations in advanced stages.

Furthermore, reductions in hip (L_M__HIP, R_M__HIP, and L_S__HIP), elbow (L_S__ELB), and knee (L_S__KNEE) were observed in Cluster 2, objectively characterizing the crouched stance typical of severely affected patients.

**Figure 7:**
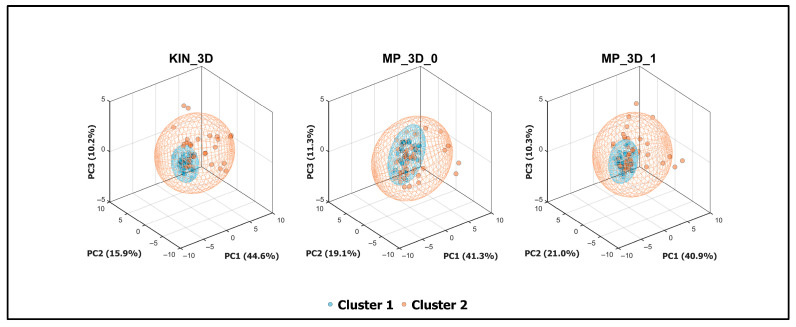
PCA components that discriminate between the 2 Clusters related to posture clinical severity across all models.

To further investigate the discriminative power of the analyzed models, a Principal Component Analysis (PCA) was performed (Figure 7). This multivariate approach captured the most significant variance associated with clinical severity in a reduced 3D space. All three models demonstrated a clear spatial separation between clusters within their respective 95% confidence ellipsoids. Notably, the variance explained by the first three principal components was remarkably consistent across models (~71–72%), suggesting that both RGB-D and RGB-only models effectively preserve the fundamental covariance structure of the postural data.

**Figure 8:**
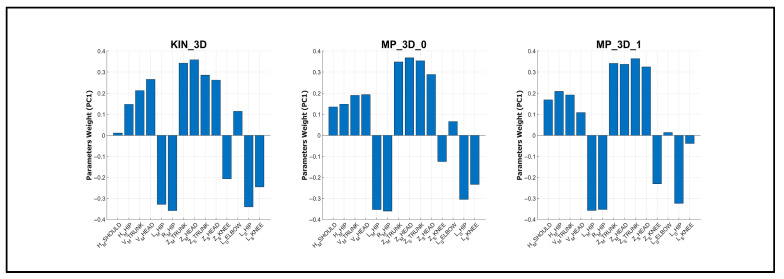
Weights of angular measurements in the first principal components (PC1) across all models.

The parameter weights on the first principal component (PC1) were analyzed to identify the primary drivers of cluster separation (Figure 8). The weight profiles were nearly identical across all models, confirming their similarities in capturing postural severity.

In conclusion, these results validate the use of markerless systems for objective postural stratification. Both RGB-D and RGB-only frameworks proved capable of detecting pathological shifts, providing a robust quantitative support to clinical assessment.

## 4. Discussion

The primary objective of this study was to investigate the potential and limitations of Google MediaPipe Pose (MP) 3D models for quantifying postural alterations and instability in PD using only RGB information. This was achieved through a synchronized dual-camera (Microsoft Azure Kinect) setup designed to overcome the inherent depth-estimation limitations of MP models and hardware-dependent algorithms of RGB-D sensors. The implemented system integrates two distinct perspectives: a main (frontal) camera and a sub (lateral) camera. This architecture allows for 3D skeletal reconstruction using both the RGB-D sensor and the RGB-only MP framework applied to standard video streams. The strength of this approach lies in its ability to directly compare the precision of a depth-measuring system (Azure Kinect) with that of a deep-learning-based estimator (Google MediaPipe). In this study, the RGB-D sensor served as the reference system, as it had previously been validated against a gold-standard motion capture system [22,26,27,63,64,65]. However, as we have ensured in our experimental protocol, it remains fundamental to avoid partial occlusions and excessive proximity between individuals within the field of view to prevent a loss of accuracy in the KIN_3D model, which could increase measurement uncertainty. Performance across three MP computational complexities was rigorously compared against the RGB-D skeletal model (KIN_3D), both from a technical and clinical perspective.

The technical results highlight that, for postural assessment in PD, higher computational complexity does not translate into greater clinical accuracy. A key finding of this study is that lower-complexity MP models achieved a high degree of technical equivalence with the KIN_3D model, particularly in assessing angular measurements and bilateral body symmetries. Conversely, the high-complexity model (MP_3D_2) introduced significant distortions in the 3D skeletal reconstruction, especially at the hip and ankle levels, despite being theoretically more sophisticated. This observation confirms our previous findings [62] on healthy participants, although some parameters of MP_3D_2 seem to perform better in the PD cohort, likely facilitated by the more pronounced abnormalities typical of PD. While the performance of MP models has proven adequate for postural analysis during relatively stable or low-dynamic tasks, MP’s performance in highly dynamic tasks (such as gait) should be evaluated, as few studies are currently available for markerless approaches [66,67,68]. While further research is still required, markerless models offer immense potential [69] for monitoring disease progression outside traditional laboratory settings, where specialized equipment such as motion capture systems remains the gold standard.

A crucial element emerging from this study is the advantage of the lateral view (Sub camera) for a comprehensive postural analysis using MP. While the frontal view is sufficient for detecting lateral trunk lean or Pisa syndrome, it systematically fails to provide the depth information required to quantify sagittal angles, such as those associated with camptocormia or knee flexion. The proposed dual-camera architecture successfully bridges this gap, elevating the MP model performance to excellent levels, comparable to KIN_3D for sagittal parameters. While a technical challenge persists at the distal extremities (knees and ankles), where the RGB-only system struggles to replicate the precision of the RGB-D sensor, the axial and proximal tracking remains highly reliable.

From a clinical perspective, one consistent finding was a strong association between elbow flexion angles and both global motor symptom severity (MDS-UPDRS III) and balance performance (BBS) across both MP and KIN_3D models. This suggests that upper-limb flexion may serve as a clinically informative surrogate marker of global motor severity; crucially, MP models were just as effective as the RGB-D sensor at intercepting this clinical indicator using only a standard video stream.

An important and clinically relevant finding concerns the dissociation between static postural alignment and dynamic locomotor performance. Our data indicate that patients maintaining a more upright frontal alignment often exhibit higher Freezing of Gait (NFOG-Q) scores and greater functional disability (MDS-UPDRS II total score). However, this association should be interpreted as hypothesis-generating and treated with caution, as it does not yet constitute clinically actionable conclusions regarding specific PD phenotypes. While awaiting confirmation in external datasets, it suggests that preserved static alignment does not necessarily reflect preserved motor function in PD. Static alignment reflects the maintenance of a stable configuration under minimal task demands, without challenging those dynamic processes (such as anticipatory postural adjustments and axial rigidity) that intervene in freezing of gait and may deteriorate independently of static posture. This interpretation is supported by evidence showing that static postural measures (specifically, center of pressure during quiet stance) do not reliably reflect dynamic gait performance [70,71]. From a measurement perspective, static angular parameters mainly capture average alignment rather than movement adaptability or postural flexibility, rendering them insensitive to the dynamic processes central to Freezing of Gait and functional performance.

Markerless postural metrics, therefore, capture structural alignment but do not directly encode dynamic adaptability or motor fluidity, underscoring the need to interpret static posture measures within a broader functional framework. The MP models successfully intercepted this complex clinical strategy with the same sensitivity as the RGB-D sensor, proving that objective postural assessment can reveal quality-of-movement information that eludes simple visual observation.

Finally, the ability of all models to discriminate between clinical severity clusters, with similar performance in PCA (Figure 7) and in detecting more relevant parameters (Figure 8), confirms that lower-complexity MP models provide a clinical stratification capability comparable to that of RGB-D sensors. While PCA-based separation could be influenced by the numerical imbalance across postural severity levels, the consistent discriminative power suggests it is primarily driven by objective kinematic patterns across all markerless models. Despite exhibiting systematic offsets, lower-complexity MP models are fully suitable for severity-impairment stratification, providing a reliable means to obtain angular metrics aligned with standardized clinical assessments. In fact, since these offsets are largely systematic and model-dependent, they do not impede the tracking of intra-patient postural trends over longitudinal monitoring. In addition, the impact of such offsets, partially due to differences in the skeletal point positioning (Figure 2), could be mitigated by adopting calibration or normalization strategies, such as referencing angular measurements to an initial neutral standing pose.

Although MP has recently been successfully utilized across various clinical settings for limb movement analysis, gait, and physical exercises, primarily using 2D models based on image coordinates [38,68,72,73,74], to our knowledge, there are no applications of 3D models in the context of Parkinson’s disease providing insights into axial postural abnormalities from both technical and clinical perspectives. To facilitate the translation of these findings into clinical practice, we have summarized the most robust and clinically relevant parameters for RGB-only postural assessment in Table 12. This table provides a simplified guideline for measuring angular deviations related to PAs, based on the technical reliability and clinical evidence presented in Table 11 and Figure 6.

Despite the promising results, several limitations must be acknowledged. First, estimating distal lower-limb kinematics (specifically at the knees and ankles) remains a challenge for RGB-only models. While the multi-view setup significantly improved tracking of sagittal angles, the inherent lack of hardware-derived depth data makes these segments more susceptible to errors caused by perspective, footwear, or slight self-occlusions during standing. To mitigate this limitation, future developments could integrate kinematic constraints, anthropometric scaling, or multi-perspective skeletal fusions, which were not included in this study, to refine and improve 3D reconstructions.

Second, the current analysis was conducted in a controlled environment. Although Google MediaPipe Pose aims to enhance accessibility, its performance in home settings and unsupervised environments, characterized by background clutter, sub-optimal lighting, and unconstrained camera angles, remains to be fully investigated.

Third, the 60 s standing task, while effective for capturing static postural alignment and compensatory rigidity, does not account for dynamic postural transitions. Future research should extend this validation to dynamic tasks (e.g., sit-to-stand or walking) to evaluate the robustness of MP models during movement.

Furthermore, while our cohort of 40 patients provided significant initial insights, the sample size needs to be expanded. A larger, more varied population would allow for more robust stratification across all MDS-UPDRS severity levels. The inclusion of patients at moderate-to-advanced stages (H&Y 2.5–4) was a deliberate decision, as postural abnormalities are more frequent and clinically evident in these stages. However, this focus limits the generalizability of our findings to early-stage PD or other populations with different postural pathologies. Future research should aim to balance the distribution of severity scores to confirm whether the high correlation coefficients and discriminative power of the angular parameters remain consistent across the full clinical spectrum, thereby enhancing both the statistical power and the clinical applicability of the results.

In addition, the observed correlations and discriminatory power provide encouraging evidence of the models’ potential, but they are intended as an exploratory analysis to illustrate how technical discrepancies impact clinical metrics. In fact, this study should be interpreted primarily as a technical, preliminary performance verification of markerless pose estimation models in a clinical postural analysis context. Future clinical studies, focusing on the diagnostic or prognostic value of these parameters, should employ larger cohorts and formal multi-comparison adjustments (e.g., False Discovery Rate) to confirm the robustness of these preliminary relationships between objective measurements and clinical scales.

Finally, a technical limitation is the current dual-camera setup, which limits the solution’s portability. While the synchronized setup was essential for direct validation of MP models against the reference KIN_3D model, the spatial requirements for positioning the Main and Sub cameras are significant. However, this hardware could be significantly simplified for obtaining similar measurements for MP by using a single standard webcam or mobile device (e.g., tablet or smartphone) and introducing a sequential assessment protocol, recording the patient first from a frontal and then a lateral perspective. This could yield the same comprehensive metrics without the burden of a multi-camera setup, further enhancing accessibility and usability.

For real-world deployment in tele-assessment and tele-rehabilitation contexts, it is currently essential to rigorously define operating conditions to ensure optimal tracking robustness and measurement reliability. This includes establishing boundaries for adequate ambient lighting (avoiding under- or overexposed conditions), appropriate clothing (avoiding excessively loose garments that may create artifacts), and adequate camera-to-patient distance to ensure full-body tracking. While these challenges can be addressed by defining strict usage requirements, future developments could integrate automated and self-adaptive mechanisms, including quality-control indicators (such as landmark confidence thresholds), occlusion detectors, and robust multi-person management procedures (when caregivers are present) to ensure tracking quality. Addressing these challenges is crucial to simplifying the effective deployment of telemedicine applications in unsupervised home settings.

## 5. Conclusions

This study demonstrates that markerless human pose estimation using lower-complexity MP models provides a reliable, hardware-independent solution for postural assessment in Parkinson’s Disease. Our findings confirm that this software-based solution, based on RGB-only information, is not only technically consistent with RGB-D cameras for several angular measurements and body symmetry metrics, but is also clinically sensitive enough to intercept complex hallmarks of the disease. In particular, MP_3D_0 represents the optimal trade-off between accuracy and computational cost: its ability to maintain high tracking consistency with clinical scales, combined with reduced processing requirements, makes it a highly suitable candidate for integration into real-time clinical monitoring systems and large-scale screening. While MP_3D_1 remains a viable option, it should be used with caution; conversely, MP_3D_2 should currently be avoided due to the significant distortions introduced during the 2D-to-3D model lifting process.

The transition from specific, short-lived hardware sensors, such as RGB-D cameras, to flexible, software-based solutions, such as Google MediaPipe Pose, marks a significant step toward scalable digital biomonitoring. By demonstrating that reliable postural assessments can be derived from standard video streams, this work supports the development of accessible, camera-based tools for precision medicine, thereby enhancing the ability to monitor PD progression and optimize therapeutic interventions in both clinical and remote settings.

## Figures and Tables

**Table 1:** Demographic and clinical characteristics of the enrolled cohort. Mean and standard deviation (SD) or number (N) are reported.

Characteristic	Value (Mean ± SD or N)
Age (years)	68.85 ± 6.29
Gender (Male/Female)	28/12
Disease Duration (years)	10.35 ± 4.67
LEDD (mg/day)	912.63 ± 413.01
Hoehn & Yahr (range: 2.5–4)	2.95 ± 0.40
MDS-UPDRS I (pts)	12.64 ± 6.04
MDS-UPDRS part II (pts)	16.53 ± 6.13
MDS-UPDRS part III (pts)	32.79 ± 13.21
MDS-UPDRS part IV (pts)	4.95 ± 4.35
MoCA (pts)	24.36 ± 3.74
UDysRS (pts)	15.11 ± 15.54
NFOG (pts)	13.69 ± 9.23
BBS (pts)	47.12 ± 8.38
PFS-16 (pts)	49.00 ± 13.66
KING (pts)	14.77 ± 15.88
PDQ-8 (pts)	9.57 ± 4.69

**Table 2:** Mean and standard deviation for horizontal angles across all 3D models ^1^. Bold values indicate no statistically significant difference (*p* ≥ 0.05) compared to the KIN_3D reference.

Parameter	MP_3D_0	MP_3D_1	MP_3D_2	KIN_3D
H_M__SHOULD (°)	**3.88 ± 0.67**	**3.86 ± 0.63**	12.19 ± 0.60 ‡	4.22 ± 0.90
H_M__HIP (°)	**2.87 ± 0.43**	**2.86 ± 0.40**	**3.36 ± 0.41**	3.04 ± 0.67
H_M__KNEE (°)	4.43 ± 1.19 ‡	7.70 ± 1.04 ‡	26.45 ± 1.00 ‡	2.81 ± 0.63
H_M__ANKLE (°)	6.36 ± 1.44 ‡	7.85 ± 1.46 ‡	9.70 ± 0.93 ‡	3.39 ± 0.94

^1^ Symbols indicate statistical significance (‡: *p* < 0.001) between the MP model and KIN_3D.

**Table 3:** Pearson’s correlation coefficients (ρ) for horizontal angles between MP models (complexity 0, 1, and 2) and KIN 3D ^1^. Bold values indicate moderate-to-strong correlations (ρ ≥ 0.5) with KIN_3D reference.

Parameter	MP_3D_0 vs. KIN_3D	MP_3D_1 vs. KIN_3D	MP_3D_2 vs. KIN_3D	MP_3D_0 vs. MP_3D_1	MP_3D_0 vs. MP_3D_2	MP_3D_1 vs. MP_3D_2
H_M__SHOULD	**0.72** **‡**	**0.72** **‡**	**0.52** **‡**	0.85 ‡	0.57 ‡	0.61 ‡
H_M__HIP	**0.73** **‡**	**0.57** **‡**	0.49 ‡	0.72 ‡	0.73 ‡	0.87 ‡
H_M__KNEE	0.13	0.22 *	−0.06	−0.02	0.18	0.65 ‡
H_M__ANKLE	−0.04	0.24 *	0.09	−0.08	−0.17	0.85 ‡

^1^ Symbols indicate statistical significance (*: *p* < 0.05, ‡: *p* < 0.001).

**Table 4:** Mean and standard deviation for vertical angles across all 3D models ^1^. Bold values indicate no statistically significant difference (*p* ≥ 0.05) compared to the KIN_3D reference.

Parameter	MP_3D_0	MP_3D_1	MP_3D_2	KIN_3D
V_M__TRUNK (°)	3.92 ± 0.55 *	3.97 ± 0.53 †	4.45 ± 0.51 ‡	3.44 ± 0.58
V_M__HEAD (°)	**12.98 ± 3.44**	**13.17 ± 2.85**	**17.58 ± 3.66**	17.69 ± 4.54
V_M__ANKLE (°)	**1.78 ± 0.41**	2.15 ± 0.43 *	**1.67 ± 0.38**	1.57 ± 0.46

^1^ Symbols indicate statistical significance (*: *p* < 0.05, †: *p* < 0.01, ‡: *p* < 0.001) between the MP model and KIN_3D.

**Table 5:** Pearson’s correlation coefficients (ρ) for vertical angles between MP models (complexity 0, 1, and 2) and KIN 3D ^1^. Bold values indicate moderate-to-strong correlations (ρ ≥ 0.5) with KIN_3D reference.

Parameter	MP_3D_0 vs. KIN_3D	MP_3D_1 vs. KIN_3D	MP_3D_2 vs. KIN_3D	MP_3D_0 vs. MP_3D_1	MP_3D_0 vs. MP_3D_2	MP_3D_1 vs. MP_3D_2
V_M__TRUNK	**0.84** **‡**	**0.87** **‡**	**0.79** **‡**	0.93 ‡	0.94 ‡	0.87 ‡
V_M__HEAD	0.45 ‡	0.44 ‡	**0.76** **‡**	0.84 ‡	0.55 ‡	0.37 †
V_M__ANKLE	0.04	0.05	0.16	0.69 ‡	0.70 ‡	0.81 ‡

^1^ Symbols indicate statistical significance (†: *p* < 0.01, ‡: *p* < 0.001).

**Table 6:** Mean and standard deviation for sagittal angles across all 3D models ^1^. Bold values indicate no statistically significant difference (*p* ≥ 0.05) compared to the KIN_3D reference.

Parameter	MP_3D_0	MP_3D_1	MP_3D_2	KIN_3D
Z_M__TRUNK (°)	109.55 ± 1.43 ‡	109.18 ± 1.23 ‡	109.52 ± 1.18 ‡	99.93 ± 1.16
Z_M__HEAD (°)	143.67 ± 1.56 ‡	143.58 ± 1.48 ‡	159.44 ± 1.11 ‡	101.40 ± 2.44
Z_M__KNEE (°)	88.04 ± 0.99 ‡	90.99 ± 1.00 †	88.33 ± 1.01 ‡	93.64 ± 0.86
Z_M__ANKLE (°)	114.59 ± 0.99 ‡	112.77 ± 1.08 ‡	113.54 ± 1.07 ‡	107.92 ± 0.83
Z_S__TRUNK (°)	87.91 ± 0.89 ‡	90.59 ± 0.99 ‡	89.58 ± 1.01 ‡	86.11 ± 1.12
Z_S__HEAD (°)	155.93 ± 1.62 ‡	157.06 ± 1.59 ‡	166.27 ± 1.74 ‡	134.48 ± 1.73
Z_S__KNEE (°)	**88.72 ± 0.96**	84.47 ± 0.79 ‡	86.87 ± 0.84 ‡	88.92 ± 0.78
Z_S__ANKLE (°)	**93.84 ± 1.05**	92.66 ± 0.91 ‡	102.90 ± 0.78 ‡	94.72 ± 0.79

^1^ Symbols indicate statistical significance (†: *p* < 0.01, ‡: *p* < 0.001) between the MP model and KIN_3D.

**Table 7:** Pearson’s correlation coefficients (ρ) for sagittal angles between MP models (complexity 0, 1, and 2) and KIN 3D ^1^. Bold values indicate moderate-to-strong correlations (ρ ≥ 0.5) with KIN_3D reference.

Parameter	MP_3D_0 vs. KIN_3D	MP_3D_1 vs. KIN_3D	MP_3D_2 vs. KIN_3D	MP_3D_0 vs. MP_3D_1	MP_3D_0 vs. MP_3D_2	MP_3D_1 vs. MP_3D_2
Z_M__TRUNK	**0.75 ‡**	**0.65 ‡**	**0.63 ‡**	0.81 ‡	0.80 ‡	0.89 ‡
Z_M__HEAD	**0.72 ‡**	**0.77 ‡**	**0.70 ‡**	0.93 ‡	0.93 ‡	0.95 ‡
Z_M__KNEE	0.17	0.28 *	0.36 †	0.66 ‡	0.49 ‡	0.76 ‡
Z_M__ANKLE	0.25 *	0.21	0.39 †	0.76 ‡	0.75 ‡	0.79 ‡
Z_S__TRUNK	**0.89 ‡**	**0.87 ‡**	**0.87 ‡**	0.98 ‡	0.98 ‡	0.98 ‡
Z_S__HEAD	**0.53 ‡**	0.46 ‡	0.30 †	0.93 ‡	0.78 ‡	0.79 ‡
Z_S__KNEE	**0.72 ‡**	**0.73 ‡**	**0.56 ‡**	0.73 ‡	0.64 ‡	0.68 ‡
Z_S__ANKLE	0.35 †	0.48 ‡	**0.65 ‡**	0.47 ‡	0.51 ‡	0.52 ‡

^1^ Symbols indicate statistical significance (*: *p* < 0.05, †: *p* < 0.01, ‡: *p* < 0.001).

**Table 8:** Mean and standard deviation for joint angles across all 3D models ^1^. Bold values indicate no statistically significant difference (*p* ≥ 0.05) compared to the KIN_3D reference.

Parameter	MP_3D_0	MP_3D_1	MP_3D_2	KIN_3D
L_M__SHOULD (°)	83.07 ± 0.70 ‡	80.90 ± 0.60 *	75.24 ± 0.51 ‡	79.98 ± 0.85
R_M__SHOULD (°)	96.40 ± 0.71 ‡	93.57 ± 0.60 ‡	92.18 ± 0.64 ‡	99.10 ± 0.85
L_M__HIP (°)	156.64 ± 1.78 ‡	**162.22 ± 1.60**	**164.80 ± 1.61**	163.46 ± 1.35
R_M__HIP (°)	156.83 ± 1.84 ‡	159.77 ± 1.76 ‡	148.02 ± 1.58 ‡	162.36 ± 1.26
L_M__KNEE (°)	153.10 ± 1.20 ‡	159.56 ± 1.20 ‡	155.05 ± 1.29 ‡	165.00 ± 1.42
R_M__KNEE (°)	153.25 ± 1.38 ‡	155.78 ± 1.53 ‡	152.06 ± 1.40 ‡	165.81 ± 1.39
L_S__ELB (°)	143.47 ± 1.78 *	148.53 ± 1.68 †	160.95 ± 1.57 ‡	145.66 ± 3.44
L_S__HIP (°)	**162.47 ± 1.82**	**162.20 ± 1.96**	168.60 ± 1.87 ‡	163.94 ± 1.25
L_S__KNEE (°)	163.01 ± 1.54 ‡	165.91 ± 1.29	157.73 ± 1.50 ‡	167.09 ± 1.30

^1^ Symbols indicate statistical significance (*: *p* < 0.05, †: *p* < 0.01, ‡: *p* < 0.001) between the MP model and KIN_3D.

**Table 9:** Pearson’s correlation coefficients (ρ) for joint angles between MP models (complexity 0, 1, and 2) and KIN 3D ^1^. Bold values indicate moderate-to-strong correlations (ρ ≥ 0.5) with KIN_3D reference.

Parameter	MP_3D_0 vs. KIN_3D	MP_3D_1 vs. KIN_3D	MP_3D_2 vs. KIN_3D	MP_3D_0 vs. MP_3D_1	MP_3D_0 vs. MP_3D_2	MP_3D_1 vs. MP_3D_2
L_M__SHOULD (°)	0.25 *	0.24 *	0.15	0.73 ‡	0.63 ‡	0.59 ‡
R_M__SHOULD (°)	0.21	0.26 *	0.02	0.60 ‡	0.66 ‡	0.54 ‡
L_M__HIP (°)	**0.51 ‡**	**0.56 ‡**	**0.56 ‡**	0.80 ‡	0.73 ‡	0.82 ‡
R_M__HIP (°)	**0.71 ‡**	**0.72 ‡**	**0.72 ‡**	0.86 ‡	0.83 ‡	0.89 ‡
L_M__KNEE (°)	−0.10	0.16	0.17	0.41 ‡	0.43 ‡	0.62 ‡
R_M__KNEE (°)	0.14	0.21	0.28 *	0.64 ‡	0.62 ‡	0.83 ‡
L_S__ELB (°)	**0.71 ‡**	**0.77 ‡**	**0.76 ‡**	0.78 ‡	0.78 ‡	0.79 ‡
L_S__HIP (°)	**0.62 ‡**	**0.71 ‡**	**0.72 ‡**	0.81 ‡	0.80 ‡	0.88 ‡
L_S__KNEE (°)	0.43 ‡	0.36 ‡	**0.52 ‡**	0.59 ‡	0.52 ‡	0.64 ‡

^1^ The symbols indicate statistically significant correlation (*: *p* < 0.05, ‡: *p* < 0.001).

**Table 10:** Systematic bias between MP models and KIN_3D for the selected postural parameters.

	MP_3D_0		MP_3D_1		MP_3D_2	
Parameter	Bias [LoA ^1^]	MAE	Bias [LoA ^1^]	MAE	Bias [LoA ^1^]	MAE
H_M__SHOULD (°)	−0.36 [−5.31–4.64]	1.99	−0.36 [−5.38–4.66]	1.95	7.97 [−0.32–16.26]	8.25
H_M__HIP (°)	−0.17 [−3.76–3.43]	1.44	−0.18 [−4.79–4.43]	1.69	0.32 [−4.93–5.57]	2.12
V_M__TRUNK (°)	0.49 [−3.30–4.26]	1.50	0.53 [−3.03–4.09]	1.45	1.02 [−3.89–5.92]	2.14
V_M__HEAD (°)	−4.70 [−48.91–39.49]	11.08	−4.52 [−48.92–39.877]	11.36	−0.11 [−32.41–32.19]	10.01
Z_S__TRUNK (°)	1.80 [−5.21–8.81]	3.37	4.48 [−3.58–12.55]	5.17	3.47 [−5.03–11.98]	4.51
Z_S__HEAD (°)	21.45 [2.35–40.55]	21.61	22.58 [1.53–43.64]	22.61	31.79 [10.26–53.33]	32.11
Z_S__KNEE (°)	−0.21 [−5.50–5.08]	2.11	−4.45 [−9.71–0.81]	4.63	−2.05 [−10.53–6.43]	3.89
L_M__HIP (°)	−6.82 [−25.67–12.03]	9.71	−1.24 [−19.00–16.53]	7.27	1.35 [−16.00–18.69]	6.71
R_M__HIP (°)	−5.53 [−20.63–9.57]	7.64	−2.59 [−17.65–12.47]	6.28	−14.34 [−29.82–1.15]	14.75
L_S__ELB (°)	−2.20 [−20.22–15.82]	7.41	2.86 [−13.29–19.01]	6.32	15.28 [−1.44–32.01]	15.41
L_S__HIP (°)	−1.47 [−16.00–13.06]	5.39	−1.74 [−17.90–14.41]	6.32	4.66 [−11.17–20.50]	7.92
L_S__KNEE (°)	−4.08 [−17.04–8.88]	6.21	−1.18 [−15.08–12.72]	5.93	−9.36 [−24.73–6.01]	10.10

^1^ LoA: Limits of Agreement.

**Table 11:** Comparison between postural severity clusters across all models ^1^. Bold values indicate statistically significant difference (*p* < 0.05) between clusters.

Parameter	MP_3D_0		MP_3D_1		KIN_3D	
	Cluster 1	Cluster 2	Cluster 1	Cluster 2	Cluster 1	Cluster 2
H_M__SHOULD (°)	3.04 ± 0.63	4.38 ± 0.70	3.55 ± 0.61	4.04 ± 0.64	3.37 ± 0.93	4.72 ± 0.88
H_M__HIP (°)	2.44 ± 0.44	3.13 ± 0.43	2.65 ± 0.40	2.99 ± 0.40	2.49 ± 0.66	3.37 ± 0.67
V_M__TRUNK (°)	3.14 ± 0.51	4.37 ± 0.58	3.05 ± 0.51	4.51 ± 0.55	2.26 ± 0.54	4.13 ± 0.61
V_M__HEAD (°)	9.87 ± 2.23	14.81 ± 4.15	10.81 ± 2.39	14.56 ± 3.12	10.48 ± 3.28	21.93 ± 5.28
Z_M__TRUNK (°)	108.91 ± 1.18	109.93 ± 1.58	107.85 ± 1.09	109.96 ± 1.31	97.45 ± 0.99	101.39 ± 1.26
Z_M__HEAD (°)	**140.53 ± 1.32**	**145.52 ± 1.70 ^†^**	**141.34 ± 1.29**	144.90 ± 1.59 *	**95.51 ± 2.10**	**104.86 ± 2.64 ^†^**
Z_S__TRUNK (°)	**85.16 ± 0.83**	**89.52 ± 0.92 ^‡^**	**87.17 ± 0.83**	**92.60 ± 1.09 ^‡^**	**82.15 ± 1.06**	**88.44 ± 1.14 ^‡^**
Z_S__HEAD (°)	**150.65 ± 1.55**	**159.03 ± 1.65 ^‡^**	**152.25 ± 1.72**	**159.89 ± 1.51 ^‡^**	131.13 ± 1.62	136.44 ± 1.80
Z_S__KNEE (°)	89.63 ± 0.86	88.18 ± 1.02	**86.15 ± 0.74**	**83.48 ± 0.81 ^†^**	**91.02 ± 0.73**	**87.69 ± 0.81 ^‡^**
L_M__HIP (°)	158.35 ± 1.50	155.63 ± 1.94	**165.25 ± 1.42**	160.43 ± 1.70 *	**167.24 ± 1.10**	**161.23 ± 1.49 ^‡^**
R_M__HIP (°)	**159.97 ± 1.61**	154.98 ± 1.98 *	**164.27 ± 1.66**	157.12 ± 1.83 *	**167.00 ± 0.98**	**159.63 ± 1.42 ^‡^**
L_S__ELB (°)	**146.72 ± 1.73**	**141.56 ± 1.82 ^†^**	151.15 ± 1.53	146.99 ± 1.76	149.02 ± 3.48	143.69 ± 3.42
L_S__HIP (°)	164.26 ± 1.58	161.41 ± 1.97	**167.37 ± 1.45**	**159.15 ± 2.26 ^†^**	**167.10 ± 0.98**	**162.08 ± 1.41 ^†^**
L_S__KNEE (°)	163.22 ± 1.30	162.89 ± 1.67	166.55 ± 1.01	165.54 ± 1.46	**171.67 ± 1.10**	**164.39 ± 1.41 ^‡^**

^1^: Symbols indicate statistical significance difference between Cluster 1 and Cluster 2 (*: *p* < 0.05, ^†^: *p* < 0.01, ^‡^: *p* < 0.001).

**Table 12:** Recommended parameters for RGB-only markerless Postural Assessment.

Clinical Target	Parameters ^1^	View	Rationale
Lateral Alterations and Postural Asymmetries	H_M__SHOULD H_M__HIP V_M__TRUNK V_M__HEAD L_M__HIP R_M__HIP	Frontal	Essential for monitoring Pisa Syndrome and lateral shifts
Sagittal Alterations	Z_M__TRUNK Z_M__HEAD Z_S__TRUNK Z_S__HEAD Z_S__KNEE L_S__HIP	Frontal/Lateral	Essential for monitoring Camptocormia and forward shifts. Measures from lateral view are preferable if available
Functional Impairments	L_S__ELB	Lateral	Associated with motor impairment and balance

^1^ Subscripts indicate the camera perspective: M for Frontal view (from Main camera), S for Lateral view (from Sub camera).

## Data Availability

The dataset is not publicly available because it is currently being used by multiple research groups funded under the same project. Data sharing will be considered once all ongoing studies within the project have been completed.

## Supplementary Materials

The following supporting information can be downloaded at: https://www.mdpi.com/article/10.3390/s26041146/s1, S1: Linear regression analysis for horizontal angles; S2: Linear regression analysis for vertical angles; S3: Linear regression analysis for sagittal angles; S4: Linear regression analysis for joint angles.
