# Supplementary material for: From RGB-D to RGB-Only: Reliability and Clinical Relevance of Markerless Skeletal Tracking for Postural Assessment in Parkinson’s Disease
Source: Sensors (Basel). 2026 Feb 10;26(4):1146. doi: 10.3390/s26041146 (PMC12944656; doi:10.3390/s26041146)
Supplement: Supplementary file 1 [file sensors-26-01146-s001.zip › Supplementary_Material.pdf]

Supplementary Appendix for:

From RGB-D to RGB-only: Reliability and Clinical Relevance of Markerless Skeletal Tracking for Postural Assessment in Parkinson's Disease

Supplementary Figures

The supplementary material presented herein highlights the performance of the analyzed markerless models across several key angular measures discussed in the main text. For each plot, the values (in degrees) obtained from the three MP models are shown, along with their respective linear regression curves, compared to the KIN_3D model. This allows for the identification of similarities and differences in the behavior of the MP models as computational complexity varies. Additionally, each plot includes Pearson's correlation coefficients (with significance levels) and the R-squared value (R^2).

S1 Horizontal angles

The figures show the differences in angular measurements across the models for horizontal shoulder and knee alignments from the frontal perspective (Main camera).

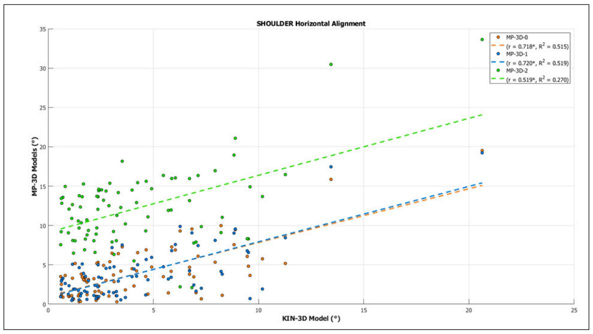

(a)

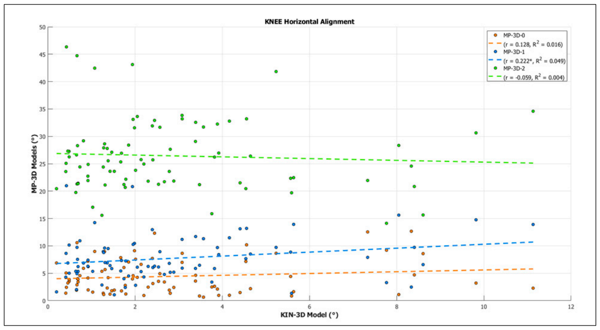

(b)

Regarding shoulder alignment, specifically the H_M_SHOULD parameter (Figure S1.a), the MP_3D_2 model shows a clear bias in angular measurements, despite maintaining significant correlations with both the lower-complexity MP models and the KIN_3D model. The other lower-complexity MP models exhibit the same behavior relative to KIN_3D. As demonstrated in our previous work on healthy subjects [1], the systematic bias introduced by MP_3D_2 arises from an incorrect reconstruction of the 3D skeletal model, leading to a distortion of shoulder alignment. This makes the MP_3D_2 model unsuitable for analyzing postural alterations, where accurately measuring shoulder alignment is crucial, as it may be associated with pathological conditions.

Regarding knee alignment, specifically the H_M_KNEE parameter (Figure S1.b), all three MP models show a substantial discrepancy compared to KIN_3D. This discrepancy increases with computational complexity, becoming particularly critical in the MP_3D_2 model. This result confirms the findings of our previous study on healthy subjects [1] and justifies the limitations of MP models in estimating the horizontal alignment of the lower limbs from a frontal perspective.

S2 Vertical angles

The figures show the differences in angular measurements across the models for vertical head and trunk alignments from the frontal perspective (Main camera).

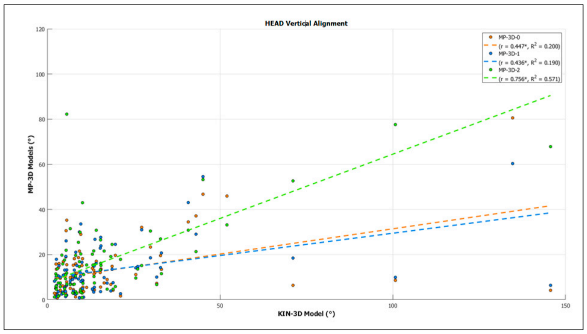

(a)

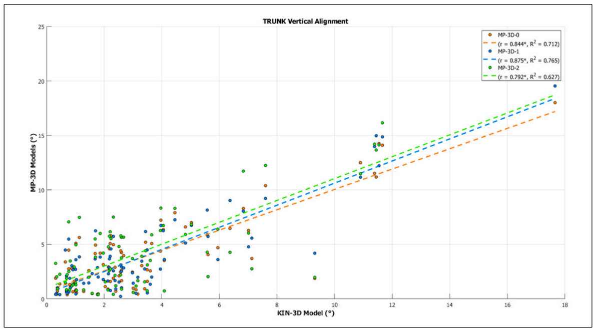

(b)

Regarding head alignment, specifically the V_M_HEAD parameter (Figure S2.a), the MP_3D_2 model diverges from the other MP models, introducing a slight bias in the measurement of head alignment relative to the shoulder center. However, it maintains greater congruence with KIN_3D. This behavior appears to contrast with what was observed in healthy subjects [1] and likely reflects the fact that the presence of pathological subjects with actual lateral postural deviations facilitates the higher-complexity MP model.

Regarding trunk alignment, specifically the V_M_TRUNK parameter (Figure S2.b), all three MP models show substantial congruence with KIN_3D and with each other. This behavior confirms the results of statistical agreement and correlation described in the main text.

S3 Sagittal angles

The figures show the differences in angular measurements across the models for sagittal head, trunk, and knee angles from the lateral perspective (Sub camera).

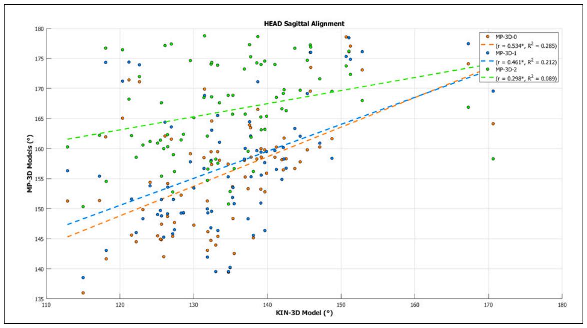

(a)

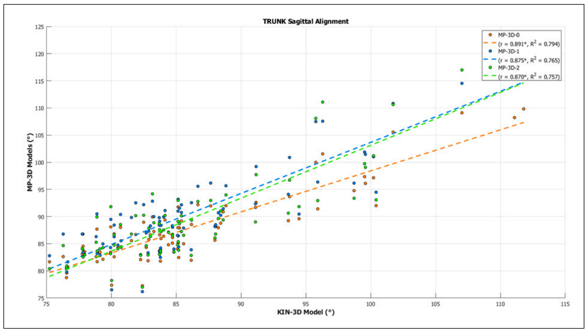

(b)

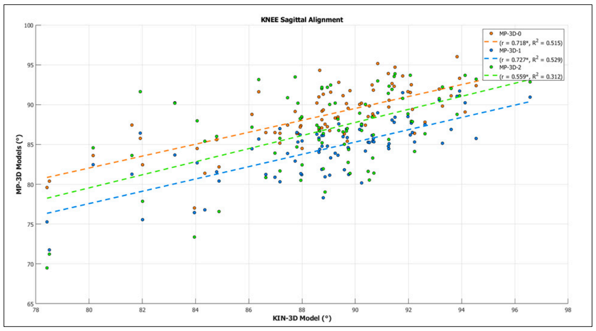

(c)

Regarding head angle, specifically the Zs_HEAD parameter (Figure S3.a), the MP_3D_2 model diverges from KIN_3D more than the other MP models, in particular for minor head angular deviations. In contrast, all MP models converge and agree with the KIN_3D model for more evident head angular alterations. As observed for the vertical angles, the MP models might also be more effective at detecting actual postural alterations of the head than in less pronounced conditions.

Regarding trunk angle, specifically the Zs_TRUNK parameter (Figure S3.b), all MP models show high congruence with each other and with KIN_3D, achieving minimal differences in trunk angular measurements and high correlation, superior to that obtained from the frontal view. In this case, the MP_3D_1 model diverges more from KIN_3D than the other MP models, though this does not appear to affect performance.

Finally, regarding knee angle, specifically the Zs_KNEE parameter (Figure S3.c), the three MP models yield very similar angular measurements, resulting in nearly parallel linear regression curves. Interestingly, the MP_3D_2 model sits between the other two complexity levels and achieves the lowest correlation with KIN_3D, likely due to greater measurement variability. In any case, the differences between the models are minimal, and the correlation values are much higher than those obtained from the frontal view, confirming the greater consistency of sagittal measurements obtainable from the lateral view.

S4 Joint angles

The figures show differences in angular measurements across the models for knee and elbow angles from the frontal (Main camera) and lateral (Sub camera) perspectives.

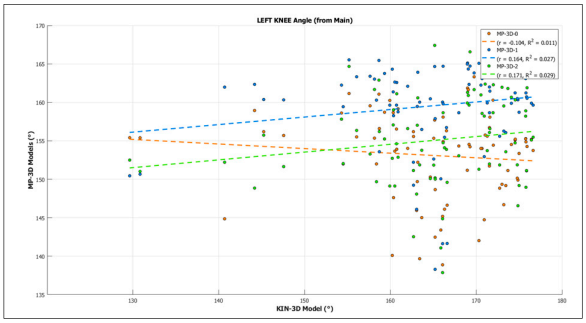

(a)

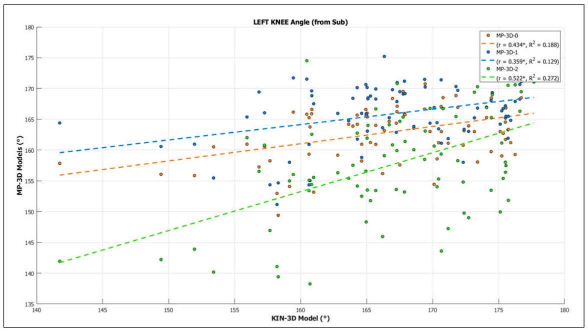

(b)

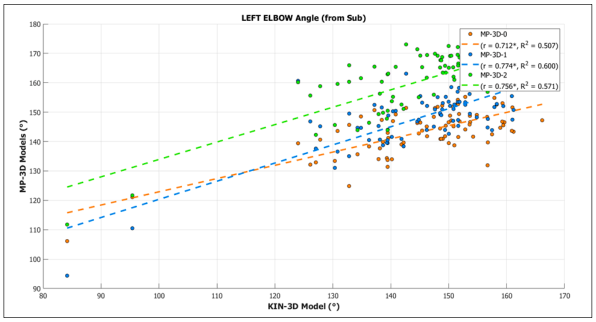

(c)

Regarding the left knee angle, a significant behavioral difference emerges between the LM_KNEE parameter measured from the frontal view (Figure S4.1) and the LS_KNEE parameter measured from the lateral view (Figure S4.2). In the former case, the MP_3D_0 model diverges from the MP_3D_1 and MP_3D_2 models, which exhibit nearly parallel linear regression curves and a constant bias. Notably, the performance of all three MP models relative to KIN_3D is superior in lateral-view measurements, with results that are congruent and highly correlated. This confirms that the lateral view supports MP models in providing more correct estimates of knee angles, which are relevant for assessing postural alterations.

Finally, regarding the left elbow angle, specifically the LS_ELB parameter (Figure S4.3), the MP_3D_0 model again diverges slightly from the other two MP models, which instead show parallel linear regression curves characterized by a nearly constant bias. The MP_3D_2 model, however, introduces an overestimation of the elbow angle (i.e., a more extended arm) compared to the other MP models, which, in contrast, result in more congruent measurements with KIN_3D. Despite this difference, the correlations between the MP models and KIN_3D are high, confirming the advantage of the lateral view in measuring elbow angles relevant to a comprehensive postural assessment.

References

[1] C. Ferraris, G. Amprimo, S. Cerfoglio, L. Vismara and V. Cimolin, "A Deep Dive Into MediaPipe Pose for Postural Assessment: A Comparative Investigation," in *IEEE Access*, vol. 13, pp. 211055-211074, 2025, doi: 10.1109/ACCESS.2025.3643126
